# Synthesis of aligned porous polyethylene glycol/silk fibroin/hydroxyapatite scaffolds for osteoinduction in bone tissue engineering

**DOI:** 10.1186/s13287-020-02024-8

**Published:** 2020-12-03

**Authors:** Yuchao Yang, Yanting Feng, Rongmei Qu, Qingtao Li, Dongming Rong, Tingyu Fan, Yiting Yang, Bing Sun, Zhenyu Bi, Asmat Ullah Khan, Ting Deng, Jingxing Dai, Jun Ouyang

**Affiliations:** 1 Guangdong Provincial Key Laboratory of Medical Biomechanics & Department of Anatomy, School of Basic Medical Science, Southern Medical University, Guangzhou, 510515 China; 2 School of Medicine, South China University of Technology, Guangzhou, 510006 China; 3 Department of Orthopedics, Zhujiang Hospital, Southern Medical University, Guangzhou, 510280 China

**Keywords:** Hydroxyapatite, Stiffness, Extracellular matrix (ECM), Bone marrow mesenchymal stem cells (BMSCs), Osteogenesis, Osteoinduction

## Abstract

**Background:**

The physical factors of the extracellular matrix have a profound influence on the differentiation behavior of mesenchymal stem cells. In this study, the effect of the biophysical microenvironment on rat bone marrow mesenchymal stem cell (BMSC) osteogenesis was studied both in vitro and in vivo.

**Methods:**

To prepare cell culture scaffolds of varying stiffness, increasing amounts of hydroxyapatite (HAp) were mixed into a polyethylene glycol/silk fibroin (PEG/SF) solution. The amount of HAp ranged from 25 to 100 mg, which provided for different ratios between HAp and the PEG/SF composite. In vitro, the effect of stiffness on the osteogenic differentiation of rat BMSCs was studied. The outcome measures, which were verified in vivo, included the protein expression of runt-related transcription factor 2 and osteocalcin, alkaline phosphatase activity, and the mRNA expression of osteogenesis-related markers.

**Results:**

Increasing amounts of HAp resulted in an increased elastic modulus of the cell culture scaffolds. The PEG/SF/HAp fabricated with HAp (50 mg) significantly increased cell adhesion and viability (*p* < 0.05) as well as the expression of all the osteogenesis-related markers (*p* < 0.05).

**Conclusions:**

We developed a novel cell culture scaffold and demonstrated that substrate stiffness influenced the osteogenic differentiation of rat BMSCs.

**Supplementary information:**

The online version contains supplementary material available at 10.1186/s13287-020-02024-8.

## Introduction

The extracellular microenvironment influences cellular behavior. Various factors within the microenvironment, including both biochemical [1, 2], and biophysical factors, play pivotal roles in cellular physiology. Biophysical factors transduce mechanical forces into biological signals resulting in the regulation of cellular behaviors [3–6]. One important biophysical factor is the extracellular matrix (ECM) stiffness, which is defined as an ability of material to undergo non-permanent deformation. Generally, ECM stiffness can influence the differentiation pathways of stem cells [7–9].

Mesenchymal stem cells (MSCs) have enormous potential for treating a wide range of diseases because of their capacity for multipotential differentiation. These cells have been shown to differentiate into bone, cartilage, muscle, fat, and a wide variety of other tissues. These findings have stimulated a great deal of interest in the field of regenerative medicine and tissue engineering [10, 11]. Tissues exhibit a wide range of stiffness (Young’s modulus) values, and it has been suggested that different mechanical properties of tissues may actively influence cell phenotypes in a tissue-specific manner [12]. A study by Engler et al. [3] demonstrated that MSCs displayed characteristics of neurogenic, myogenic, or osteogenic phenotypes after being cultured on hydrogel substrates mimicking the stiffness of neural, muscle, or bone tissue, respectively. Therefore, the goal of many research efforts has been to mimic stem cell microenvironments to evaluate the importance of physical cues for stem cells [13, 14].

Studies have shown that extracellular matrix stiffness is mainly through the interaction between integrin regulating cell differentiation, while boosting mineralization can increase RUNX2 and the expression of COL1A1 [15], at the same time can also be as the main medium of osteogenesis differentiation under mechanical stimulation, and play a key role through the focal adhesion kinase (FAK)/extracellular related kinases (ERK) and MAPK signaling pathways. Activation of PI3K is also important in mediating osteogenic differentiation because it is involved in the regulation of MSC proliferation and osteogenic differentiation, but the differentiation [16] and selection mechanism of stem cells is still unclear. In addition, integrins regulate cell differentiation through the WNT signaling pathway, which enhances the expression of integrin mRNA and protein and further regulates osteogenic differentiation [17, 18]. Other studies have indicated that mechanical force is an important factor in TAZ/YAP activities [19]. Stiff ECM stimulates the nuclear localization of TAZ/YAP and promotes osteogenic differentiation, while soft ECM inhibits its nuclear localization and induces lipid differentiation. Rigid ECM activates Rho GTPase, which stimulates F-actin aggregation and activates TAZ and YAP [20]. Appropriate physical factors can induce osteogenic differentiation in stem cells by stimulating them to secrete large amounts of collagen, through Notch, WNT, BMP signaling pathways [9, 21] such as OCN and OPG, BSP, and other important proteins involved in osteogenesis by playing essential role in osteogenic differentiation bone mineralization and bone formation in different stages [22].

To achieve this goal, the use of various biomaterials has been explored for tissue engineering applications in vitro and implantation in vivo. Some studies developed discrete gradient scaffolds by fabrication of two or three phases separately, which are subsequently integrated with suturing, glue, or press-fitting [23]. Recently 3D printing methods have been used for the osteochondral (OC) scaffold fabrication due to their ability to fabricate interconnected porous scaffolds with well-controlled pore geometries; the scaffold structure may be designed to exhibit appropriate mechanical properties that match the host tissue [24]. Specifically, for bone tissue engineering, these materials consist of bioactive ceramic, bioactive glass, and biological or synthetic polymers with mineralization and incorporation of silk protein or decellularized native bone [25, 26]. Native bones are mainly composed of an organic phase including type I collagen and inorganic minerals, with specific mechanical properties [27]. Increasingly, a major research focus in bone tissue engineering has been the manufacture of scaffolds with the appropriate properties. These properties, which imitate the natural ECM microenvironment, include scaffold porosity and interconnectivity. This enables cell adhesion and infiltration of growth factors into spaces in the scaffold.

The goal of this study was to characterize the effects of cell culture scaffold stiffness on rat bone marrow mesenchymal stem cell (BMSC) osteogenesis. The scaffolds were fabricated from polyethylene glycol/silk fibroin/hydroxyapatite (PEG/SF/HAp) composites. Polyethylene glycol (PEG) was chosen as the backbone of the scaffolds because its crosslinking density can be modulated [28]. Moreover, it is one of the few biocompatible synthetic polymers approved by the US Food and Drug Administration (FDA) for biomedical applications [29]. Hydroxyapatite (HAp) is found in bones and can increase the stiffness of scaffolds, enabling the mechanical properties and osteoinductive effects to be controlled. Silk fibroin (SF) has also been used in bone tissue engineering because of its controllable degradation rates, excellent biocompatibility, and induction of a subdued inflammatory response [30, 31]. First, we evaluated the morphology and biocompatibility of rat BMSCs cultured on a PEG/SF/HAp scaffold. Next, in both in vitro and in vivo assays, we evaluated the alkaline phosphatase (ALP) activity, immunofluorescence staining of osteogenic-related proteins, and mRNA expression of osteogenesis-related genes. These genes included runt-related transcription factor 2 (RUNX2), osteoprotegerin (OPG), osteocalcin (OCN), and bone sialoprotein (BSP).

## Materials and methods

### Materials

Poly (ethylene glycol) diacrylate (PEGDA), hydroxyapatite (HAp), LiBr, APS, TEMED, FITC, and DAPI were purchased from Sigma-Aldrich (USA). Phalloidin, DiD dye, and live/dead kit were purchased from ThermoFisher Scientific. All other chemicals were purchased from Sigma-Aldrich unless otherwise indicated. RUNX2, OCN, OPG, and BSP antibodies were purchased from Santa Cruz (USA). The raw silks were obtained from Simatech Co. (JiangSu, China).

### Scaffold fabrication

Cocoons from the mulberry silkworm *Bombyx mori* were boiled for 30 min in 0.02 M Na_2_CO_3_ solution and then rinsed thoroughly with double-distilled water to extract the glue-like sericin proteins. Next, the extracted silk proteins were dried briefly at 37 °C for 1 h. The resulting silk fibroin (SF) was then dissolved in 9.3 M LiBr solution at 60 °C for 4 h. The SF solution was dialyzed against distilled water with a dialysis membrane (12 kDa) for 3 days with a continuous renewal of the distilled water. The dialysate was centrifuged (5000 rpm; 10 min at 4 °C) to remove the impurities and aggregates formed during dialysis. The final concentration of the SF aqueous solution was 7–8% (wt/vol), which was determined by weighing dried films at 60 °C.

Next, 60 mg PEGDA (molecular weight, 700 kDa) was dissolved in 500 μL of distilled water to form a solution with a predetermined concentration (12% wt/vol) at room temperature. The solution was cooled to 0 °C, followed by the addition of SF (38.6 μg), four different amounts of HAp in separate tubes (25, 50, 75, or 100 mg), ammonium persulfate (APS; 20% aqueous solution, 15 μL), and tetramethylethylenediamine (TEMED; 3 μL). In all four groups, the solution was poured into a polypropylene tube (diameter, 8 mm; height, 20 mm), which was kept close to the surface of frozen isopropyl alcohol (− 80 °C) [32, 33]. After completely freezing each solution, the solidified samples were transferred into a freezer to complete the polymerization process at − 20 °C for 12 h. Then, dry unidirectional cryogels were prepared using a freeze dryer for 24 h until they reached a constant weight. After this, the cryogels were immersed in excess distilled water at ambient temperature for 48 h. The water was replaced every 4 h to remove the unreacted materials and obtain the final cryogel.

### Scanning electron microscopy (SEM)

SEM (Hitachi S-3000N) was performed to determine the scaffold pore structure and surface topography. In brief, scaffolds were washed in phosphate-buffered saline (PBS), fixed in 2% glutaraldehyde overnight, washed in alcohol (with a strength up to 70%), and then freeze-dried overnight in a lyophilizer. To prepare the samples for SEM, the freeze-dried scaffold samples were coated with gold for 10 min in advance and then cut in both the parallel and perpendicular directions to the freezing direction.

### Fluorescein isothiocyanate (FITC) staining

To observe the morphology of the scaffold during swelling, FITC (a fluorescent probe) was selected to stain the cryogel samples: FITC was dissolved in NaOH aqueous solution to form a solution (5 mg/L). The resulting cryogel samples were cut into slices with a thickness of approximately 0.5 mm and immersed in the FITC solution. After 30 min, the stained samples were washed with PBS until no dye was detected in the discarded PBS. Then, the scaffolds were observed using an optical microscope (Olympus BX51, Japan).

### Scaffold characterization

#### Fourier-transform infrared (FTIR) spectrometry

FTIR spectra of PEG/SF/HAp with different proportions of HAp were characterized with a spectrometer (Bruker VERTEX 33, Germany). Peaks at different wavenumbers correspond to particular linkages and functional groups; therefore, spectra from 4000 to 400 cm^− 1^ were recorded. The presence of different polymers in the cryogel blend, possible bonds, and crosslinking were confirmed by FTIR. The FTIR spectra of pure PEG, SF, and HAp were measured as controls.

#### Thermogravimetric analysis (TGA)

The thermal stability of the PEG/SF/HAp scaffolds was examined using TGA. To determine the weight loss of the PEG/SF/HAp scaffolds at different temperatures, TGA measurements were performed up to 1000 °C (at a heating rate was 10 °C/min) in a nitrogen atmosphere using the STA449F5 instrument (Netzsch, Germany).

#### X-ray diffraction (XRD)

The crystal structure was determined by XRD using an Empyrean diffractometer system (PANalytical) and Cu K_α_ radiation (with a wavelength of 1.5406 $\dot{\mathrm{A}}$). Data were collected for 2θ values of 20–90°, with a step width of 0.02° and a counting time of 1 s per step.

### Mechanical properties of cryogels

Using a dynamic mechanical analyzer (Bose 3220-AT Series II), Young’s modulus (compressive elastic modulus) of the cryogels was measured parallel to the freezing direction. Young’s modulus (*E*, kPa) represents the slope of a straight line fitted for the stress-strain curves in the range from 5 to 15% strain. The E was calculated based on the following equation: *E* = Δσ/Δε, where Δσ is the tensile stress and Δε is the tensile strain. The fully swollen (in PBS) cryogel columns were cut into cubes with a length of approximately 8 mm. They were then saturated with 0.1 M PBS (pH 7.4) and subjected to unconfined compression. The saturated samples were compressed to as much as 50% of their original length by placing them between two arms of the dynamic mechanical analyzer (DMA) at room temperature. The speed of compression between the two parallel plates was 1 mm/min.

### Swelling ratio (SR) of the cryogels

Dry cryogels, which had been prepared using a freeze dryer for 24 h (until they reached a constant weight), were incubated in distilled water, and the weight of the swollen cryogels was measured at regular time intervals. The SR of the cryogels was calculated based on the following equation: SR = WS/WD, where WS is the weight of the swollen cryogel, and WD is the original weight of the dry cryogel.

### Rat BMSC isolation and seeding

All animal experiments were approved by the Southern Medical University Animal Ethics Committee and were performed in compliance with the regulations for the use of rat BMSCs. Fresh bone marrow aspirates were obtained from the upper femurs and tibias of 4-week-old Sprague Dawley (SD) rats, and BMSCs were isolated and characterized as previously described [34]. Briefly, the BMSCs were expanded in growth medium (Dulbecco’s modified Eagle medium (DMEM)/F12 (Gibco, USA)) supplemented with 10% fetal bovine serum (FBS, Gibco, USA) and 1% penicillin-streptomycin (Gibco, USA). The BMSCs were cultured up to the third passage and then seeded on the scaffolds (with 3 × 10^7^ cells/mL of scaffold) in growth medium without any osteogenic factors, for 21 days. A 40-μL aliquot of BMSC suspension was pipetted onto the scaffolds and allowed to percolate through. For 2 h, the scaffolds were flipped 180° every 20 min and 10 μL of growth medium was added every 20 min to prevent the cells from drying out.

### Live/dead staining

For the cell imaging assay, cells were stained with a Live/Dead Cell Imaging kit, according to the manufacturer’s instructions (ThermoFisher Scientific) and imaged on Olympus BX51 to determine whether they were live (green, ex/em 488 nm/515 nm) or dead (red, ex/em 570 nm/602 nm).

### Alkaline phosphatase (ALP) staining

Rat BMSCs (1 × 10^4^ cells/cm^2^) were seeded onto the scaffolds and cultured in growth medium. After 7 and 14 days, ALP activity was assayed using a 5-bromo-4-chloro-3-indolyl phosphate (BCIP)/nitro blue tetrazolium (NBT) ALP color development kit (Beyotime Institute of Biotechnology, China). The ALP-positive area was measured using Image J software for three randomly selected fields under an optical microscope (Olympus BX51, Japan).

### Immunofluorescence staining

After being cultured on the scaffolds for 7, 14, and 21 days, the cells were washed three times with DMEM and fixed in 4% paraformaldehyde for 10 min at 37 °C. Cells were then washed with PBS three times and treated with 0.1% (v/v) Triton X-100 in PBS at 4 °C for 10 min to increase the permeability of the cell membranes. After three additional washes with PBS, the samples were incubated in 2% BSA in PBS at 37 °C for 30 min to block non-specific interactions. The cells were then incubated overnight at 4 °C with a rabbit monoclonal antibody against RUNX2 (1:200, mouse) or OCN (1:200, mouse) (Santa Cruz, USA). After being washed twice in PBS, they were further stained with a fluorescent-labeled secondary antibody (1:200) (Beyotime, China) and phalloidin (Excitation/Emission: 495/518 nm)/DiD (Excitation/Emission: 644/665 nm) respectively for 1 h and 4′,6-diamidino-2-phenylindole (DAPI, 1:500) at room temperature for 0.5 h, followed by three washes with PBS. The cells were observed using a confocal laser scanning microscope (C2, Nikon, Japan). The images obtained were further analyzed using Image J software (National Institutes of Health, USA).

### RNA isolation and real-time quantitative PCR (RT-qPCR)

The mRNA expression levels of important osteogenesis-related genes, including *OCN*, *OPG*, *BSP*, and *RUNX2*, were determined by RT-qPCR at 7, 14, and 21 days after culturing on the scaffolds. Total RNA was isolated and purified using an RNA extraction kit (Tiangen, China) according to the manufacturer’s instructions. First-strand cDNAs were generated by reverse transcription of 1 mg total RNA with a RevertAid First Strand cDNA Synthesis Kit (Thermo Scientific). RT-qPCR was performed in triplicate with an ABI Step One Plus System (Applied Biosystems, USA) and a fluorescence-labeled SYBR Green/ROX qPCR Master Mix Kit (Thermo Scientific). A melting curve analysis was used to confirm the PCR specificity, and the data were analyzed using SOS 2.1 software (Applied Biosystems). The relative expression levels were analyzed using the 2^−ΔΔCt^ method and normalized to those of the housekeeping gene glyceraldehyde 3-phosphate dehydrogenase (*GAPDH*). Calibration was performed using BMSC growth on the four PEG/SF/HAp scaffolds at 7, 14, and 21 days. The forward and reverse primer sequences for RT-qPCR of each gene were as follows:

- *GAPDH*: F-AGGTCGGTGTGAACGGATTTG
  R-TGTAGACCATGTAGTTGAGGTCA
- *OCN*: F-ATTGTGACGAGCTAGCGGAC
  R-TCGAGTCCTGGAGAGTAGCC
- *OPG*: F-CAGTGTGCAACGGCATATCG
  R-CCAGGCAAGCTCTCCATCAA.
- *BSP*: F-AGCTGACCAGTTATGGCACC
  R-TTCCCCATACTCAACCGTGC
- *RUNX2*: F-CAACCGAGTCAGTGAGTGCT
  R-AAGAGGCTGTTTGACGCCAT

### Application of BMSC-laden PEG/SF/HAp scaffolds for bone regeneration in vivo

The BMSC-laden PEG/SF/HAp scaffolds were investigated in a rat calvarial defect model. Two groups were designed as follows: namely unladen (Group I) and laden (Group II) with BMSCs of PEG/SF/HAp scaffolds implanted into rats skull defects. Autograft bone was implanted into the calvarial defect as the positive control (*n* = 6), and the calvarial defects were created without any treatment as the negative control (*n* = 6). PEG/SF/HAp scaffolds with different magnitudes of stiffness (laden with or without BMSCs) were implanted into parietal bone defects (*n* = 6) as the experimental group. Seventy-two SD rats (weight range 180–220 g) were anesthetized (0.2 mL/100 g b.w. chloral hydrate). Two 5-mm-diameter circular acute calvarial bone defects were created in each specimen, scalp skin was incised, and the periosteum was removed to visualize the skull. A 5-mm-diameter cylindrical defect was created on each side of the parietal bone (without any damage to the dura mater) using a dental bur, under irrigation with sterile saline solution (Fig. 5A (a)). The circular bone plug was removed gently, every two different PEG/SF/HAp scaffolds (*n* = 3) laden with or without BMSCs was placed on each side of the rat skull, each rat being its own control (Fig. 5A (b)). Calvarial defects created in the skulls were left empty as negative controls and autografts were positive controls (*n* = 3). Wound closure was achieved by a two-layer suturing (periosteum, skin) using absorbable sutures. Immediate post-operative care included intraperitoneal injection of penicillin to prevent infection and buprenorphine (0.02 mg/kg b.w.) for analgesia. The animals were fed with a standard diet and housed individually under standard conditions. Wound healing progressed without any sign of infection, material exposure, or other complication.

### Micro-CT examination of samples

Eight and 12 weeks after the operation, samples were taken from the rats at the corresponding time points. All the rats were anesthetized intraperitoneal with 10% chloral hydrate (0.2 mL/100 g), and the rats were sacrificed by cervical dislocation after satisfactory anesthesia. The skin, subcutaneous tissue, and periosteum were cut in the middle of the head to fully expose the skull, and the surrounding soft tissue was fully separated. The cranial was completely removed along the edge of the parietal bone, so as not to damage the tissue at the defect site. Muscle and periosteal tissue on the surface of the cranium were removed. After the blood was washed in PBS, the cranial cap specimens were placed in a sterile 50-mL centrifuge tube for preservation and placed in a refrigerator at − 20 °C for storage. After collection, micro-CT scanning was performed.

For bone regeneration analysis, calvarial specimens were obtained from euthanized rats and scanned using a micro-CT to observe the morphological features of the defect site. The micro-CT device was set at 85 kV and 135 mA, and a tomographic rotation of 180°. The results were reconstructed with Mimics 20.0 software.

### Biomechanical analysis of regenerated tissue

At 8 and 12 weeks post-operation, samples from the HAp (25, 50, 75, and 100 mg) groups, blank control group, and autologous bone graft group were collected. An ElectroForce 3510 Bose instrument was used to conduct a biomechanical test on the defect site, and a stress-strain curve was obtained. The loading speed of the biomechanical instrument was set at 3 mm/min, and the maximum load on the regeneration tissue was defined according to the stress-strain curve of the vertices.

### Histological analysis

Regenerated tissue samples obtained from the defect were washed with PBS, fixed in 4% phosphate-buffered formalin (pH = 7.4) for 48 h, and then decalcified with 20% EDTA decalcification solution. The solution was changed every 3 days and decalcified for 4 weeks. Decalcification is terminated when the skull tissue becomes soft, the needle is easy to penetrate, and the skull becomes homogeneous and transparent and subsequently dehydrated, transparent, waxed, embedded, and sliced. After dewaxing, rehydration, and hematoxylin-eosin staining, the sections obtained from the middle of the specimen were photographed for analysis.

### Statistical analysis

At least three independent experiments were carried out. Results are reported as mean ± standard deviation (SD), and the means were compared between groups using Student’s *t* tests. The level of statistical significance was set at *P* < 0.05.

## Results

### Morphology of the PEG/SF/HAp scaffolds

**Fig. 1:**
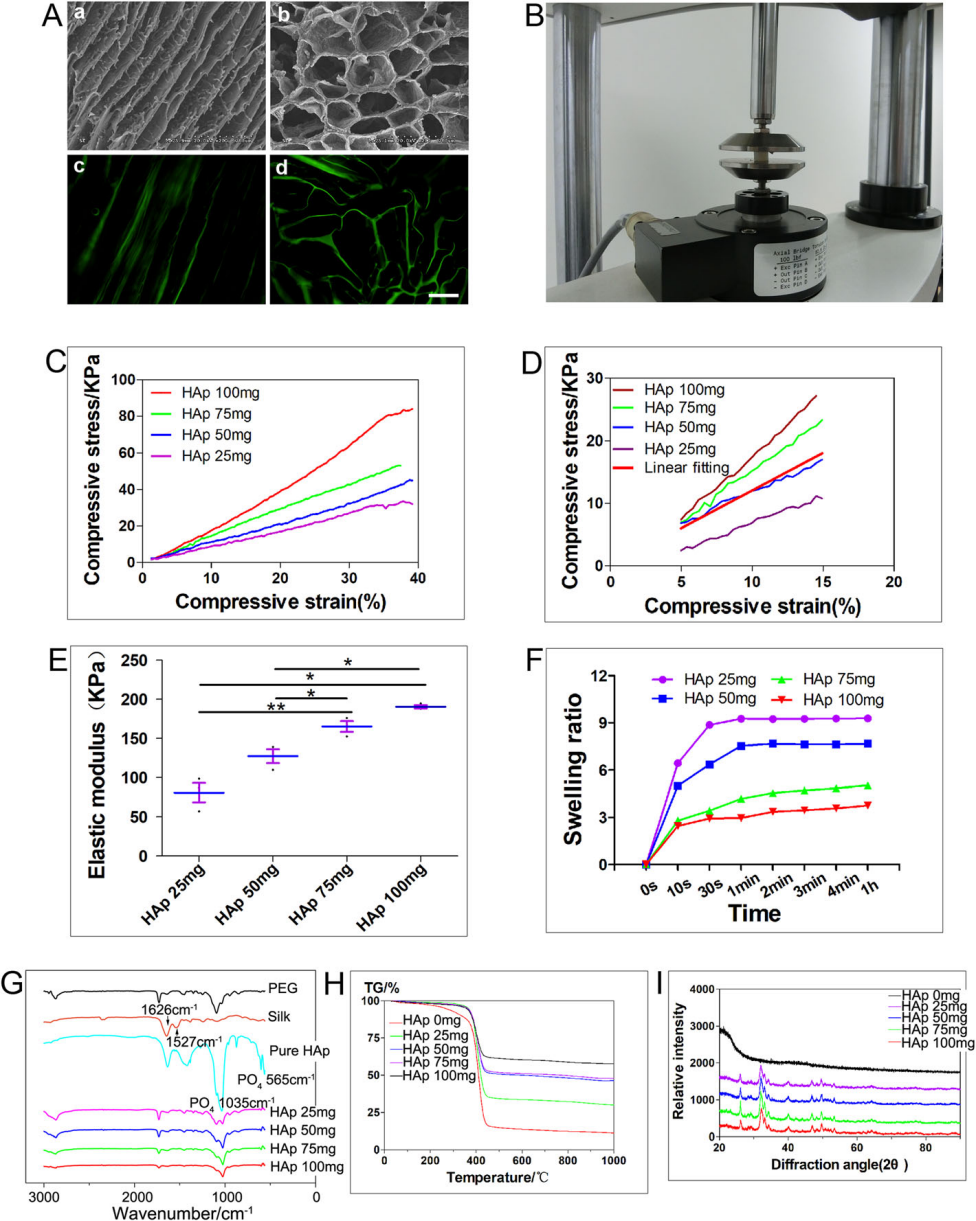
Physical properties of PEG/SF/HAp scaffolds. **A** SEM images (a and b) and FITC staining images (c and d) of the scaffolds prepared by unidirectional freezing. (a and c) Longitudinal sections parallel to the freezing direction. (b and d) Cross-sections perpendicular to the freezing direction (scale bar = 200 μm). **B** Diagram showing compression using a dynamic mechanical analyzer (DMA). **C** Original stress-strain curves of the scaffold. **D** Stress-strain curves (colorful) and fitted straight lines (red, just display one) from 5 to 15% strain. **E** Young’s modulus of the scaffolds with different concentration of hydroxyapatite (HAp). **F** Swelling kinetics of the scaffolds prepared with different proportions of HAp. **G** Fourier-transform infrared (FTIR) spectra of pure PEG, silk fibroin (SF), hydroxyapatite (HAp), and PEG/SF/HAp scaffolds (25, 50, 75, and 100 mg). **H** Thermogravimetric analysis (TGA) curve comparisons of PEG/SF/HAp scaffolds with different proportions of HAp. **I** X-ray diffraction (XRD) analysis of PEG/SF/HAp scaffolds

**Fig. 2:**
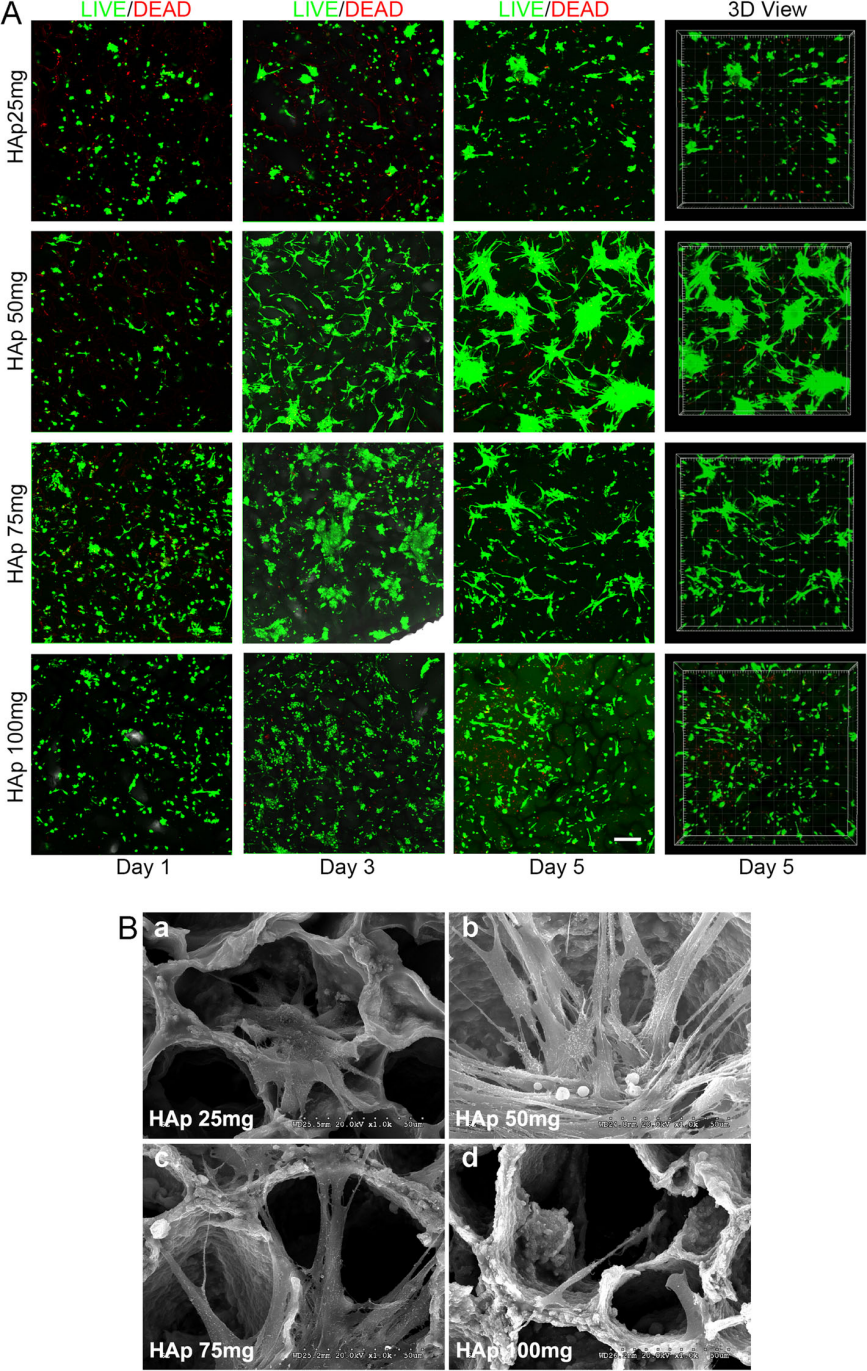
Biocompatibility of scaffold materials. **A** LIVE/DEAD assays of BMSCs cultured on PEG/SF/HAp scaffolds with different magnitudes of stiffness at 1, 3, and 5 days, based on 2D and 3D views (scale bar = 200 μm). **B** SEM images of BMSCs cultured on the PEG/SF/HAp scaffolds for 5 days (scale bar = 50 μm)

A PEG/SF/HAp scaffold was constructed from a PEGDA 700 aqueous solution (12% wt/vol) with isopropyl alcohol as the cooling agent. The morphology (including the pore structures) of the scaffold, prepared by unidirectional freeze-drying, as examined by SEM and fluorescence microscopy, revealed a longitudinal view parallel to the freezing direction and a cross-sectional view perpendicular to the freezing direction (Fig. 1A). The uniform structure could be observed clearly in the SEM images, which show rough-walled micro-pipes with ridges parallel to the freezing direction. SEM images indicated that there were no significant differences in the structure and morphology of the scaffolds consisting of different HAp concentrations. Despite this, there was improved homogeneous distribution of HAp particles in HAp (25, 50, and 75 mg) scaffolds compared with the HAp (100 mg) scaffold (Fig. S1). The high-magnification images of the PEG/SF/HAp scaffolds showed visible HAp particles embedded in the wall of pipes compared with that in scaffolds without HAp (Fig. S2). Furthermore, BMSCs cultured on the scaffolds showed excellent alignment during growth in the longitudinal section (Fig. S3). The BMSCs also spread around and stretched into the pore in the cross-section (Fig. 2B).

### Mechanical properties of the scaffolds

Each scaffold was compressed parallel to the freezing direction using a dynamic mechanical analyzer (DMA) (Fig. 1B). The typical stress-strain curves of the single compression test for the scaffolds containing different amounts of HAp are shown in Fig. 1C. Figure 1C shows the original stress-strain curves, whereas Fig. 1D shows a magnified image of the stress-strain curves (from 5 to 15% strain), as well as a fitted straight line for these curves. Figure 1E demonstrates that the Young’s modulus of the scaffolds from approximately 80.98–190.51 kPa (Supplemental Table 1). The HAp concentration of the PEG/SF/HAp scaffolds influenced the stiffness. By varying the amount of HAp used in the synthesis steps, PEG/SF-based scaffolds with four different proportions of HAp (25, 50, 75, and 100 mg) and different magnitudes of stiffness (80.98 to 190.51 kPa) were successfully prepared.

### Swelling ratio (SR) of the scaffolds

The swelling kinetics of the scaffolds with different proportions of HAp are shown in Fig. 1F. The four samples reached their equilibrium swollen state after incubation in distilled water for 1 h, and this was correlated with the crosslinking density of the polymer networks. The swelling behavior occurred because water molecules diffused easily into the interior of the scaffolds. This is due, in part, to the abundant porous structure of the scaffolds prepared using unidirectional freezing. Our findings demonstrated that the equilibrium SR matched well with the pore diameter of the scaffolds.

### Chemical characterization of PEG/SF/HAp scaffolds

FTIR, XRD, and TGA were used to characterize the PEG/SF/HAp scaffolds. The FTIR spectra of pure PEG, SF, and HAp, along with PEG/SF/HAp (25, 50, 75, and 100 mg) are shown in Fig. 1G. Regarding pure SF, a β-sheet structure was formed with gelation, as indicated by the major FTIR bands at 1625 cm^− 1^ in the amide I region and at 1527 cm^− 1^ in the amide II region. The phosphate groups in pure HAp showed characteristic FTIR bands between 900–1100 cm^− 1^ and 550–600 cm^− 1^ (Fig. 1G). FTIR spectra of the HAp (25, 50, 75, and 100 mg) PEG/SF/HAp scaffolds revealed bands at 900–1100 cm^− 1^ and 550–600 cm^− 1^ (Fig. 1G). These results suggest that the structure of the PEG/SF/HAp composite consists of β-sheet crystallites embedded in an amorphous matrix. The pure PEG demonstrated no characteristic absorption band in both the amide I and II regions, which means that it had a negligible effect on the structural properties.

The TGA curves of composite scaffolds indicated differences in their thermal behavior. The TGA curves obtained for the PEG/SF/HAp scaffolds with different proportions of HAp are shown in Fig. 1H. The curves reveal that a slight weight loss took place from about 65–200 °C because of the release of water. This weight loss began at a lower temperature for the scaffolds with higher proportions of HAp. This is because HAp acts as a dehydrating agent and accelerates the oxidative stabilization reaction. For all PEG/SF/HAp scaffolds, the majority of the weight loss was observed between 260 and 390 °C and was attributed to the pyrolysis of the PEG/SF/HAp scaffolds. The percentage of weight loss caused by the pyrolysis process was smaller when the proportion of HAp was high. The remaining weight loss occurred from 600 to 800 °C, and it was greater when the proportion of HAp was lower.

XRD mapping of calcium elements was used to characterize the PEG/SF/HAp scaffolds, specifically the HAp particle deposits observed on the scaffold surface. For the HAp (25, 50, 75, and 100 mg) PEG/SF/HAp scaffolds (Fig. 1I), a major 2θ reflection peak at around 26° represented (002) diffraction, and another at approximately 32° represented the overlapped diffractions of (211), (300), and (202). This indicates that the introduction of crystalline properties into the amorphous structure of the PEG/SF scaffolds was due to the presence of HAp.

### Identification and characterization of rat BMSCs

Rat BMSCs were isolated from the femurs and tibias of 4-week-old male SD rats. At the initial passage, the cells were heterogenous, and this heterogeneity increased with subsequent passaging. After three passages, most cells exhibited mesenchymal morphology. These cells were negative for markers CD31 and CD45 and expressed the rat BMSCs surface markers CD54 and CD90 (Fig. S5).

### Cell viability

LIVE/DEAD assay (Molecular Probes, Thermo Fisher) was performed after cells were seeded on the scaffolds. After being cultured on the scaffolds for 1, 3, and 5 days, the BMSC morphology changed from round to spindle shape. Cells in the HAp (50 mg) group displayed better viability than other groups, suggesting that the PEG/SF/HAp scaffold containing HAp (50 mg) has good cytocompatibility (Fig. 2A, SM1–SM4 on day 5). Additionally, the SEM images indicate that BMSCs that spread well on the scaffolds exhibited spindle morphology and good adhesion ability. Additionally, a large number of pseudopods adhered to the pore wall of the scaffold, especially in the HAp (50 mg) group (Fig. 2B).

### Immunofluorescence staining

**Fig. 3:**
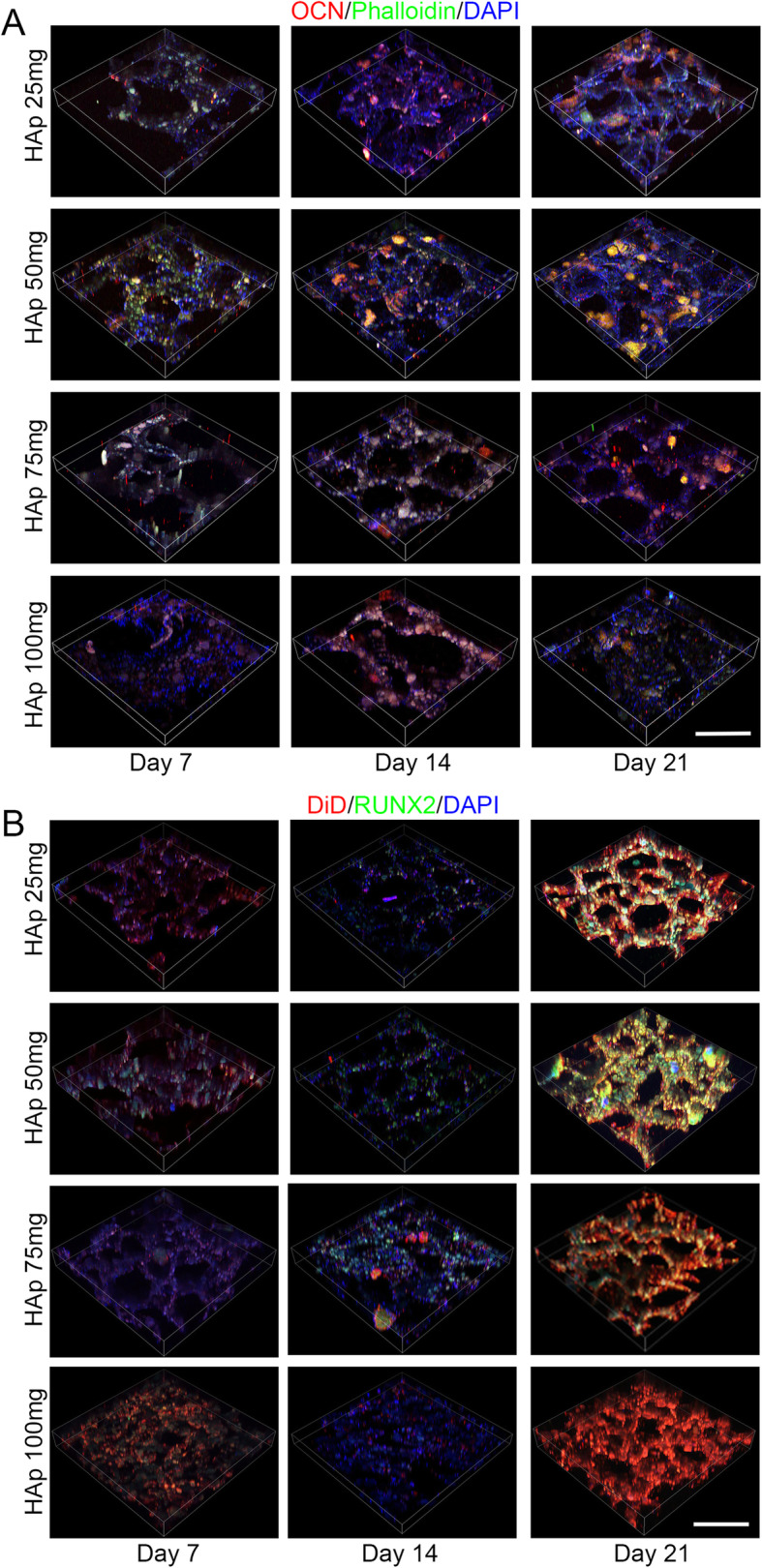
Osteogenic properties of PEG/SF/HAp scaffolds. Immunofluorescence staining of osteocalcin (OCN) (**a**) and runt-related transcription factor 2 (RUNX2) (**b**) in BMSCs cultured on the scaffolds for 7, 14, and 21 days, based on a 3D view (scale bar = 100 μm)

To examine the osteogenic differentiation of BMSCs on scaffolds with different magnitudes of stiffness, we analyzed the expression of the osteogenesis-related protein OCN and RUNX2 by immunofluorescence after 7, 14, and 21 days of culturing in growth medium (Fig. 3). Figure 3 shows higher OCN and RUNX2 expression in the HAp (50 mg) group than in the other groups, in both the 2D (Fig. S4) and 3D view (Fig. 3). This indicates that BMSCs on the HAp (50 mg) group are well spread out and tend to undergo osteogenesis at a higher rate, based on the protein expression of the osteogenesis-related markers OCN and RUNX2.

### Alkaline phosphatase (ALP) staining

**Fig. 4:**
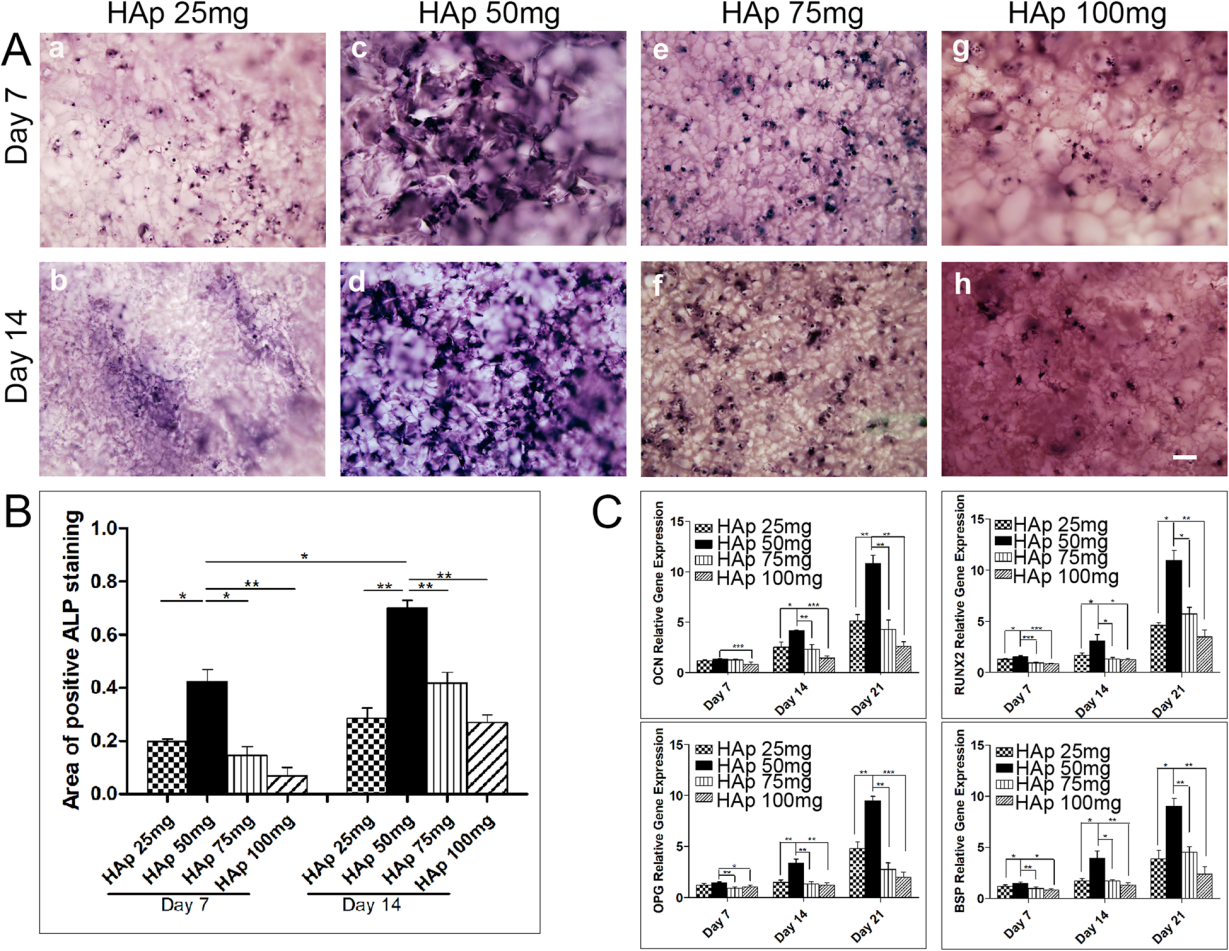
**A** ALP staining of rat BMSCs cultured on scaffolds without osteogenic induced media for 7 and 14 days (scale bar = 200 μm). **B** Semi-quantitative ALP staining data were analyzed by Image J software. **C** Osteogenesis-related mRNA expression (bone sialoprotein, BSP; osteocalcin, OCN; osteoprotegerin, OPG; runt-related transcription factor 2, RUNX2) of BMSCs after 7, 14, and 21 days of culture on different scaffolds

To further study the effect of scaffold stiffness on the osteogenic differentiation of BMSCs, we examined another osteogenesis-related marker. ALP is an essential osteogenesis-related marker in the early stage of osteogenesis. A higher ALP expression was observed in the HAp (50 mg) group than in the other groups after 14 days of culture (Fig. 4A). Semi-quantitative analysis indicated significant differences between the HAP (50 mg) group and the other groups (25, 75, and 100 mg; *p* < 0.05; Fig. 4B).

### Relative expression of osteogenesis-related markers in vitro

To examine the effect of the PEG/SF/HAp scaffolds on the osteogenic differentiation of BMSCs in vitro, RT-qPCR was conducted to investigate BSP, OCN, OPG, and RUNX2 mRNA expression in the four groups. These factors play an important role in all stages of bone formation, including osteogenic differentiation, biomineralization, remodeling, and maintenance. BSP, OCN, OPG, and RUNX2 expression was low at day 7 (Fig. 4C). At days 14 and 21, BSP, OCN, OPG, and RUNX2 expression was higher in the HAp (50 mg) group than in the other groups, with increases of three to four times compared with the HAp (100 mg) group (*p* < 0.05 vs. other conditions).

### In vivo study

#### Macroscopic observation of the regeneration of calvarial defects

**Fig. 5:**
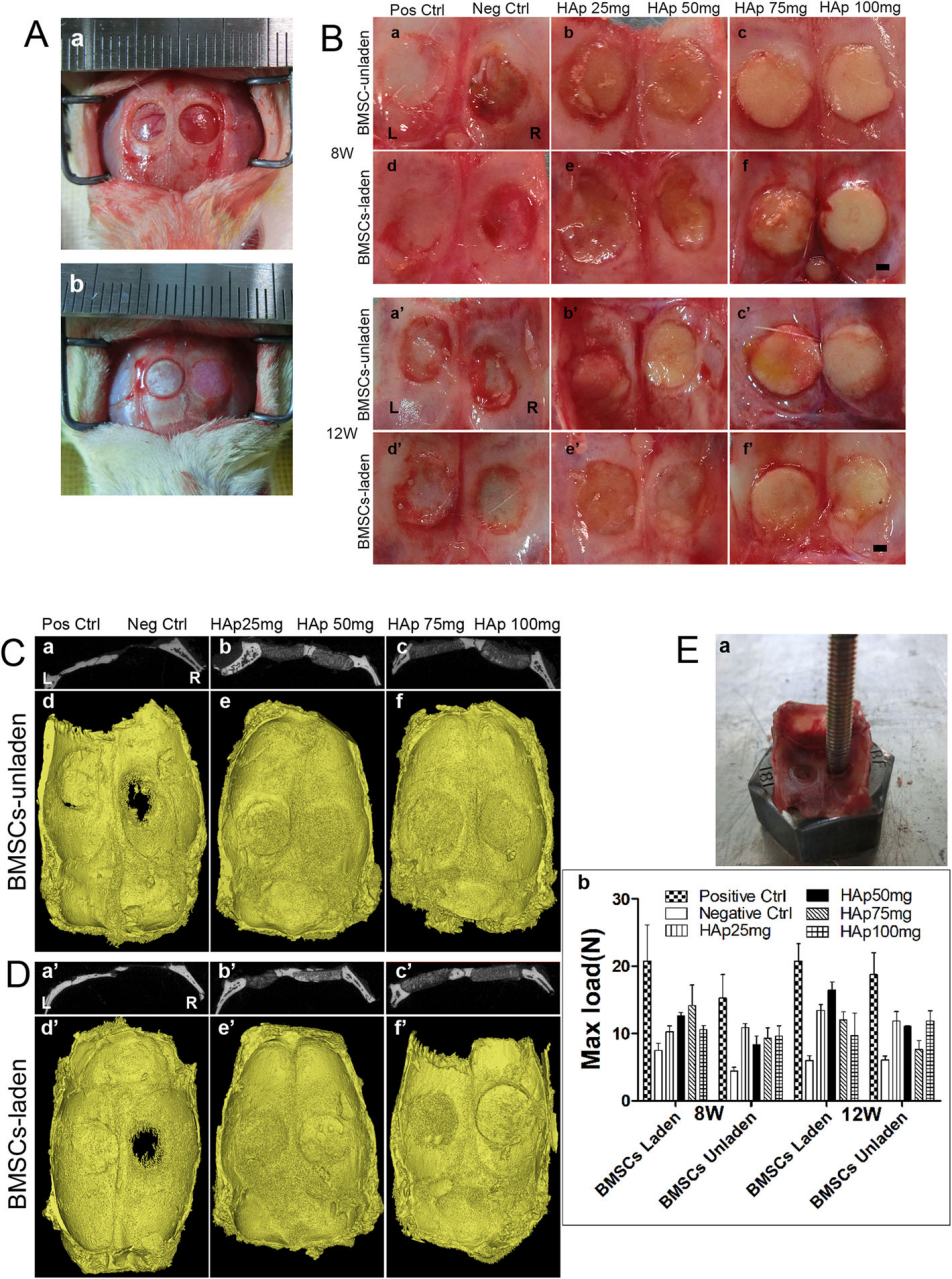
Establishment of the rat calvarial defect model and implantation of the different scaffolds into the defect. **A** (a) The calvarial defect model, where the diameter of the calvarial defect is 5 mm. (b) Autograft bone and PEG/SF/HAp scaffold were implanted into the calvarial defect. **B** The morphology of the regenerated tissues in the calvarial defect area after 8 and 12 weeks (inside view, scale bar = 1 mm). (a–c and a’–c’) BMSC-unladen positive control group, negative control group, HAp 25 mg group, HAp 50 mg group, HAp 75 mg group, and HAp 100 mg group, respectively. (d–f and d’–f’) BMSC-laden positive control group, negative control group, HAp 25 mg, HAp 50 mg, HAp 75 mg, and HAp 100 mg groups. **C** (a–c) Gray value of CT image of coronal plane calvarial defect repaired specimen. (d–f) 3D reconstruction images of micro-CT scanning data of the samples after 12 weeks of calvarial defect model by PEG/SF/HAp scaffold implanted with unladen BMSCs (inside view). **D** (a’–c’) Gray value of CT image of coronal plane calvarial defect repaired specimen. (d’–f’) 3D reconstruction images of Micro-CT scanning data of the samples after 12 weeks of calvarial defect model by PEG/SF/HAp scaffold implanted with laden BMSCs (inside view). **E** Biomechanical properties of regenerated tissue. (a) Biomechanical testing process. (b) The maximum load force applied to the calvarial defect area repaired by scaffolds

The tissue-implant constructs were retrieved at 8 and 12 weeks post-operation (Figs. 5B and S6A). The surfaces of the areas with defects were covered with new tissues, which can be seen in each implantation group (laden or unladen with BMSCs). Although the defect areas in the positive control group were filled entirely, new bone formation was minimal in the negative control group (Figs. 5B and S6A). All the defect areas of the scaffold-implanted groups were covered with new tissue and closely connected with the surrounding area. In all the PEG/SF/HAp scaffold implantation groups, HAp (50 mg) group laden with BMSCs exhibited higher bone calcium deposition (Fig. 5B (e’), R). Further, the defect area was covered by regenerated bone at 12 weeks, indicating good osteointegration ability of the HAp (50 mg) scaffold. This was significantly superior to that of other groups (unladen or laden) with BMSCs. Also, there was no apparent calcium deposition and formation of new bone tissue in the HAp (75 and 100 mg) implantation group at 12 weeks.

#### Micro-CT scanning of the regenerated tissue

Twelve weeks post-operation, all animals were sacrificed for micro-CT scanning. The calvarial defect in the positive group was filled with autograft bone and integrated with the surrounding host bone (Fig. 5C, D). In the negative group, the large cavity defect was clearly visible, indicating that there had been little bone regeneration. When compared with the 3D reconstructed images of the tissue-implant constructs with unladen BMSCs, the scaffolds laden with BMSCs were found to display relatively better bone formation and bone density. In particular, in the HAp (50 mg) group laden with BMSCs, the defect area was filled with new bone tissue, and the bone density of the newly formed bone was similar to that of the host bone (Fig. 5D (b’), R). This indicates that the HAp (50 mg) scaffold laden with BMSCs showed the best performance in terms of inducing osteogenesis.

#### Biomechanical analysis of regeneration tissue

After 8 and 12 weeks of implantation, the regeneration tissue specimens were retrieved and placed on the test nut for direct mechanical testing (Fig. 5E (a)). The loading head was applied to the implants with gradually increasing stress until the implants were pushed out. Figure 5E (b) showed that the maximum load of all the implantation groups increased with the implantation time, indicating that the degree of bone integration increased over time. At week 12, the maximum load was increased dramatically in the BMSC-laden HAp (50 mg) group. These data showed that the BMSC-laden HAp (50 mg) group had the best osteointegration and biomechanical properties during bone formation.

#### Histological analysis of the regenerated tissues

All samples were collected, and three regenerated samples from the calvarial defects were selected from each group for histological section staining.

##### H&E staining

**Fig. 6:**
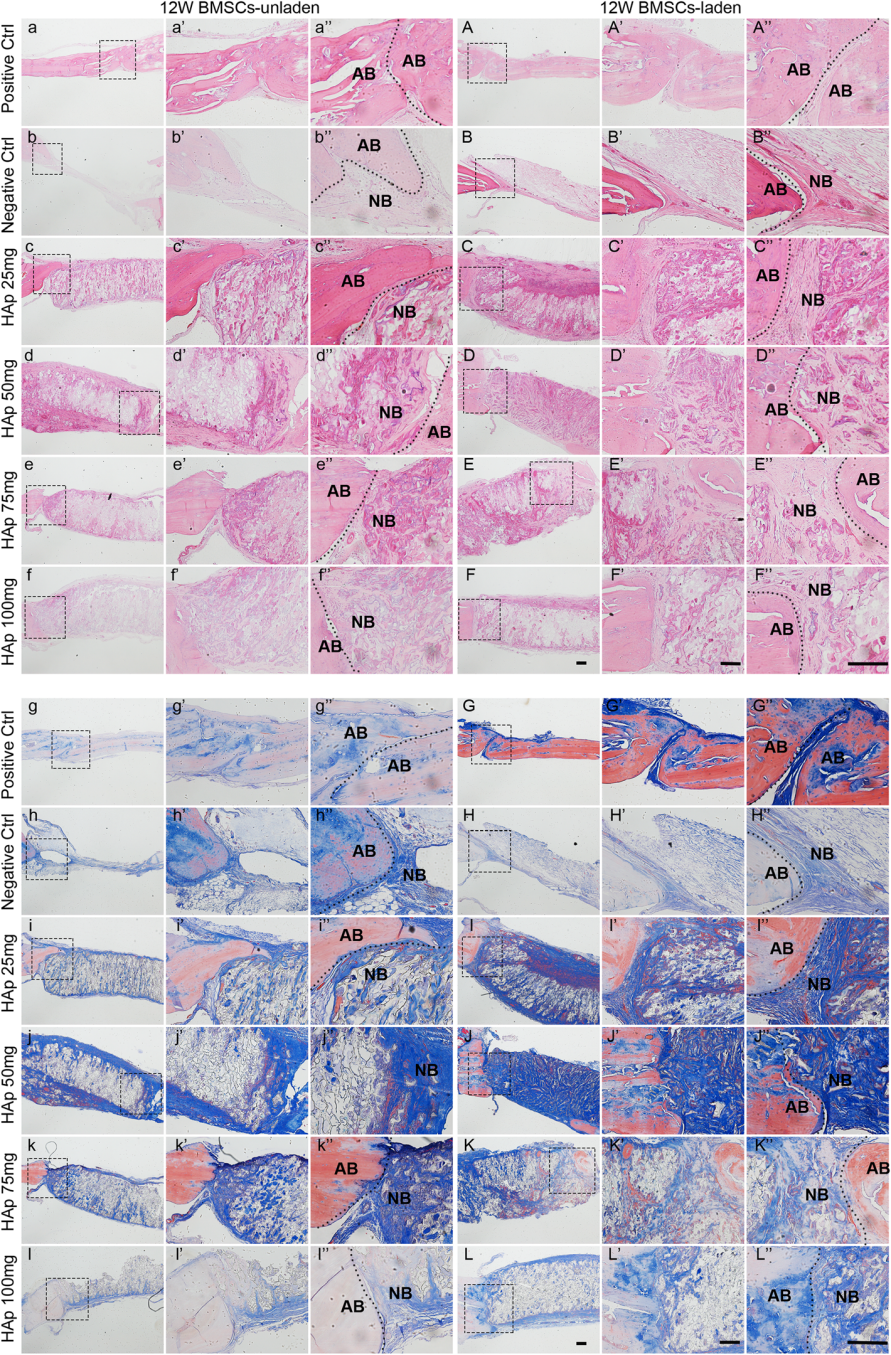
Histologic staining of calvarial defect repair specimens. H&E and Masson trichrome staining of specimens in each group unladen or laden with BMSCs, and AB is autogenous bone and NB is new bone (scale bar = 200 μm)

As shown in Fig. S7 a-f” and Fig. 6a–f” (see full morphology of H&E staining in Fig. S11 a-f), in the regenerated samples from the calvarial defect unladen with BMSCs, the defect area in the positive control group was filled with red-stained, homogeneous tissue of the same density. In the negative control group, only a small amount of loose and low-density tissues filled the defect, and there were only a few stained nuclei. At 8 and 12 weeks, in all groups with unladen BMSCs, the defect areas had few uniform, dense new tissues, but a newly formed thin layer of bone tissue could be seen in the HAp (50 mg) group (Fig. S7 d-d” and Fig. 6d–d”). However, all PEG/SF/HAp scaffold implantation groups laden with BMSCs, which had more uniform and dense new tissue, showed better regeneration compared with those with unladen BMSCs (Fig. S8 a-f” and Fig. 6A–F”, see full morphology of H&E staining in Fig. S11 A-F). The defect areas filled with a thick layer of newly formed bone tissue, which presented lamellar and relatively complete bone integration. Overall, we observed large amounts of regenerated tissue, and a thicker newly formed bone layer where the HAp (50 mg) scaffold was implanted (Fig. 6D–D”). The new bone in this group was the most similar to the original host bone.

##### Masson trichrome staining

In Masson trichrome staining experiments, collagen was stained blue. Collagen accounts for 90% of bone proteins in natural bone and is therefore an indicator of regenerated bone. At 8 and 12 weeks, the defect area in the positive group was filled with blue-stained tissues and limited collagen in the regenerated tissue in negative group (Fig. S9 a-b” and Fig. 6g–h”, see full morphology of Masson staining in Fig. S12 a-b). In Fig. S9 c-f” and Fig. 6i–l” (see full morphology of Masson staining in Fig. S12 c-f), the regions implanted with HAp (25, 75 mg and 100 mg) scaffolds showed a few more uniform and blue-stained regenerated tissues and a thin layer of new bone tissue. Additionally, many small lacunae were generated by the degradation of the scaffolds and low distribution of chondroblast cells. In the HAp (50 mg) group, there was more homogeneous, dense, and blue-stained tissue formation in the defect area of the cranial and bilateral side. The thicker layer of newly formed bone tissue grew into the middle of the defect area, and there were fewer small lacunae after scaffold degradation. As shown in each group laden with BMSCs (Fig. S10 c-f” and Fig. 6I–L”, see full morphology of Masson staining in Fig. S12 C-F), a large number of new tissues filled in the defect areas where the PEG/SF/HAp scaffolds with different magnitudes of stiffness had been implanted, and these had a more beneficial effect compared with unladen BMSCs. And in terms of these defect areas, denser, blue-stained tissues filled with a thick bone layer. In the HAp (50 mg) group, the majority of the defect area was filled by a large number of newly formed thick bone layers, and the tissue was lamellar with relatively complete bone integration. Hence, the histological analysis of bone formation further confirms that the HAp (50 mg) group promoted bone formation most efficiently compared with the other groups.

## Discussion

Although a large number of studies have examined the effect of ECM stiffness on stem cell behavior, the relationship between ECM stiffness and changes in cell morphology, adhesion, proliferation, and differentiation remains to be fully understood. Mechanotransduction is the process by which cells perceive mechanical stimuli and convert it into biochemical signals. This process regulates several aspects of cellular behavior in a time-dependent manner, including migration, proliferation, and differentiation. Some studies have shown that both the behavior and fate of cells are determined not by a single signal, but by a complex network of signals operating at different time and length scales [35]. Elucidation of the interaction between cells and biomaterials, or ECM, may contribute to the clinical application of new biomaterials for tissue regeneration.

In general, cells are subjected to a combination of external and internal forces that activate specific intracellular signaling pathways. These forces can also be transferred to other cells through intercellular connections or via extracellular matrix adhesions. The ECM stiffness regulates the amplitude of these forces, thereby affecting the intracellular signaling pathways, and stronger forces promote MSC osteogenic differentiation [3]. This mechanism usually involves focal adhesions, mechanical sensors, and nuclear signaling factors that cause changes in the gene and protein expression profiles [36]. Through interactions between integrins and the ECM, the cell provides a direct link between the cell and the environment. Integrins combine the targets in the extracellular space with the focal adhesion domain (through the cytoplasmic domain and cytoskeletal structure). These adhesion sites, which consist of multiple protein complexes, enable mechanical coupling to link cells and the extracellular matrix. The adhesion force is stable on hard substrates and produces less tension on the soft substrate, which leads to unstable adhesions [37–39]. This viscoelasticity allows the activation of a wide variety of signaling pathways through its plasticity, allowing the cell to respond precisely to the forces exerted [40]. As a result, tuning the ECM stiffness to optimize osteogenic differentiation may aid in the repair of bone defects. Additionally, this method overcomes the limitations of traditional methods for the regeneration of autogenous bone and repair of allograft bone defects by serving as a bone tissue engineering material that is convenient to construct, with self-renewal and differentiation capacity. In particular, bone marrow mesenchymal stem cells are easy to source and their use is not limited by ethical issues; therefore, these represent one of the most promising seed cells [41].

In this study, the aligned porous PEG/SF/HAp scaffolds with different magnitudes of stiffness were used to support cell adhesion and proliferation. Through co-culture with BMSCs, the effect of ECM stiffness on BMSCs osteogenic differentiation and bone defect repair was investigated. The live/dead staining results for BMSCs grown on biomaterials (Fig. 2A) and SEM images (Fig. 2B) were observed to promote the adhesion and proliferation of stem cells. BMSCs have a strong ability to regenerate and self-renew, and can differentiate into osteoblasts under appropriate conditions to promote the repair and regeneration of bone tissue. These behaviors also require the survival of stem cells in an adaptive microenvironment that stimulates their differentiation [42]. As a result of the very porous structure of PEG/SF/HAp scaffold material, water molecules can easily spread into the scaffold and expand it. The scaffold can maintain higher water content and obtain sufficient nutrients for cell growth. In addition, the scaffold itself has specific mechanical properties, and the stiffness of the scaffold itself could promote BMSC differentiation into osteoblasts. These factors combined provide an appropriate 3D microenvironment and the required temporal and spatial gradient necessary for the growth and differentiation of BMSCs.

Based on in vivo and in vitro experimental results, we found that the HAp (50 mg) scaffold laden with BMSCs promoted osteogenesis-related protein and gene expression better than the other groups. For example, ALP is one of the important and relevant markers of early osteogenesis within 14 days. The expression of ALP in the HAp (50 mg) group was the highest compared with the higher stiffness scaffolds fabricated from HAp (75 and 100 mg) where expression decreased (Fig. 4A–B). A similar trend was observed in osteogenesis-related protein and gene expression (Fig. 4C). This finding indicates that the expression of osteogenesis-related proteins or genes do not increase with HAp concentration or increasing stiffness. Meanwhile, at 8 and 12 weeks, regenerated specimens from each group showed that PEG/SF/HAp scaffold laden with BMSCs containing HAp (50 mg) was used to repair rat calvarial defects (Fig. 5B and Fig. S6A) much more effectively compared with other scaffold groups laden with BMSCs and all groups unladen with BMSCs.

H&E staining of the skull defect repair specimens showed that the collagen was stained red, and the nuclei were stained blue. Type I collagen is the main component of bone tissue. The collagen structure is dense and even, as evidenced by H&E staining. The HAp (50 mg) scaffold group laden with BMSCs demonstrated better repair. The overall staining showed that the new bone was most similar to the normal bone tissue, but the lamellar structure was not as apparent as in the normal bone and probably required further remodeling (Fig. 6). The same effect could be observed from the Masson trichrome staining (Fig. 6). This indicated that the PEG/SF/HAp scaffold containing HAp (50 mg) and laden with BMSCs had the best overall effect on the osteogenic differentiation of stem cells and the repair of skull defects in rats.

**Fig. 7:**
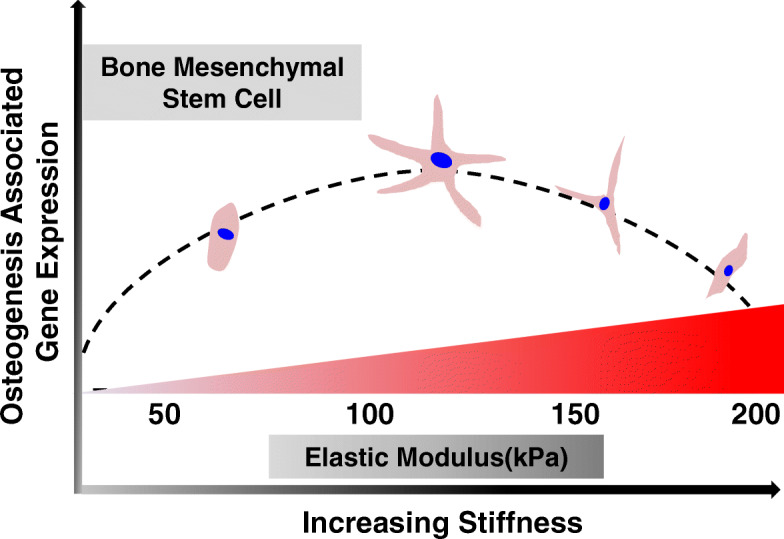
Schematic of the effect of different magnitudes of ECM stiffness on BMSC differentiation through regulation of the expression of osteogenesis-related genes

Osteogenic differentiation of BMSCs is dependent on substrate stiffness within a specific range (Fig. 7). Studies have shown that the increased stiffness of the ECM promotes the expression of several cytoskeleton-related proteins, including vinculin, and the activation of the YAP/TAZ signaling pathway [43]. TAZ and YAP are also known as effector proteins in the Hippo signaling pathway, which plays an important role in cell proliferation, tumorigenesis, and stem cell self-renewal. YAP protein is transferred to the nucleus after phosphorylation and is involved in the expression of osteogenesis-related genes and proteins. When the ECM stiffness is excessively high, the ability of the cell to sense the mechanics of the microenvironment decreases. Therefore, it is unable to transduce the excessive mechanical signaling to the relevant proteins on the cell membrane, and this leads to a decrease in the osteogenic differentiation ability [19]. TAZ stimulates Runx2 target genes but inhibits PPARγ-mediated gene transcription [20].

The osteogenic marker RUNX2 is the upstream regulatory gene encoding stem cells that differentiate into osteoblasts. It plays an essential role in osteogenic differentiation and bone formation and participates in signaling pathways such as Notch, Wnt, and BMP [9, 21]. High expression in the HAp (50 mg) group indicated that the osteogenic differentiation of BMSCs occurred under a specific suitable range of ECM stiffness. Osteoblasts secrete OCN, OPG, and BSP, which are characteristic components of the bone matrix. These components play an important role in osteogenic differentiation, bone mineralization, bone repair, and other stages of bone formation [22]. The increased expression of these osteogenic-related genes indicates that HAp (50 mg) scaffold has a significant effect on the osteogenic differentiation of BMSCs induced by scaffold stiffness.

Elucidation of the effect of mechanical factors on the differentiation of stem cells is necessary to develop methods for simulating the ECM microenvironment, in order to obtain an environment that closely mimics the physiological or pathological state of the body and provides cells with a more realistic 3D environment for survival. Incorporating spatial and temporal effects, cell growth transforms from the 2D to the 3D environment, and both cell-cell and cell-ECM connections and signal transduction are enhanced. Cells perceive the physical and chemical properties of the ECM through receptors on the cell membrane and convert chemical and mechanical signals into biological signals within cells. This process initiates a series of transcription and translation events. At the same time, activated cells can transmit this stimulation to neighboring cells through cell-cell connections that activate corresponding signaling pathways of neighboring cells.

Multiple findings within this study indicate that the PEG/SF/HAp scaffolds containing HAp (50 mg) are biocompatible and have appropriate biomechanical properties. Additionally, the stiffness of these scaffolds can induce osteogenic differentiation in BMSCs in vitro and promote better calvarial defect repair in vivo by modulating gene and protein expression levels.

## Conclusions

In this study, we successfully constructed PEG/SF/HAp scaffolds, with different magnitudes of stiffness, which had compositional and aligned porous structural features that mimicked the natural bone ECM. These scaffolds supported BMSC adhesion, spread, viability, and proliferation. The results showed that the PEG/SF/HAp scaffold has excellent biocompatibility and biomechanical properties, as well as an outstanding ability to induce osteogenic differentiation. PEG/SF/HAp scaffolds can induce osteogenic differentiation of BMSCs in vitro by modulating gene and protein levels. The insights into the effects of substrate stiffness are useful for understanding the differentiation behaviors of BMSCs within a complex microenvironment and can be used to guide the design of biomaterials for controlling stem cell fate.

## Supplementary Information

**Additional file 1: Figure S1.** SEM images of the structure and morphology of the scaffolds without HAp and with different HAp concentrations (scale bar = 500 μm).

**Additional file 2: Figure S2.** High-magnification SEM images (right) of the scaffolds without HAp and with different HAp concentrations.

**Additional file 3: Figure S3.** BMSC culture on the scaffold showing alignment during growth in the parallel section, based on the 2D view (a–d) and 3D view (e–h). (scale bar = 200 μm).

**Additional file 4: Figure S4.** Immunofluorescence staining of osteocalcin (OCN) (A) and runt-related transcription factor 2 (RUNX2) (B) in BMSCs cultured for 7, 14, and 21 days on the scaffolds, based on the 2D view (scale bar = 100 μm).

**Additional file 5: Figure S5.** Surface molecular profile of BMSCs.

**Additional file 6: Figure S6.** A. The morphology of the regenerated tissues in the calvarial defect area after 8 and 12 weeks (outside view, scale bar = 1 mm). a-c and a’-c’ were BMSCs-unladen positive control group, negative control group, HAp 25 mg group, HAp 50 mg group, HAp 75 mg group, and HAp 100 mg group respectively. d-f and d’-f’ were BMSCs-laden positive control group, negative control group, HAp 25 mg, HAp 50 mg, HAp 75 mg, and HAp 100 mg groups. B. Three-dimensional reconstruction images of micro-CT scanning data of the samples after 12 weeks of calvarial defect model by PEG/SF/HAp scaffold implanted with unladen (a-c) and laden (a’-c’) BMSCs (outside view).

**Additional file 7: Figure S7.** H&E staining of specimens in each group unladen with BMSCs at 8 weeks (scale bar = 200 μm).

**Additional file 8: Figure S8.** H&E staining of specimens in each group laden with BMSCs at 8 weeks (scale bar = 200 μm).

**Additional file 9: Figure S9.** Masson trichrome staining of specimens in each group unladen with BMSCs at 8 weeks (scale bar = 200 μm).

**Additional file 10: Figure S10.** Masson trichrome staining of specimens in each group laden with BMSCs at 8 weeks (scale bar = 200 μm).

**Additional file 11: Figure S11.** Full morphology of H&E staining of specimens in each group unladen and laden with BMSCs at 12 weeks (scale bar = 500 μm).

**Additional file 12: Figure S12.** Full morphology of Masson staining of specimens in each group unladen and laden with BMSCs at 12 weeks (scale bar = 500 μm).

**Additional file 13: Movie 1.** Three-dimensional reconstruction of BMSC culture on PEG/SF/HAp scaffold with 25 mg hydroxyapatite (HAp).

**Additional file 14: Movie 2.** Three-dimensional reconstruction of BMSC culture on PEG/SF/HAp scaffold with 50 mg hydroxyapatite (HAp).

**Additional file 15: Movie 3.** Three-dimensional reconstruction of BMSC culture on PEG/SF/HAp scaffold with 75 mg hydroxyapatite (HAp).

**Additional file 16: Movie 4.** Three-dimensional reconstruction of BMSC culture on PEG/SF/HAp scaffold with 100 mg hydroxyapatite (HAp).

**Additional file 17: Table S1.** Young’s modulus of the scaffolds with different concentration of hydroxyapatite (HAp).

## Data Availability

All the supporting data can be downloaded.

## Abbreviations

MSCs: Mesenchymal stem cells
BMSC: Bone marrow mesenchymal stem cell
HAp: Hydroxyapatite
PEG: Polyethylene glycol
SF: Silk fibroin
RUNX2: Runt-related transcription factor 2
OCN: Osteocalcin
ALP: Alkaline phosphatase
ECM: Extracellular matrix
OPG: Osteoprotegerin
BSP: Bone sialoprotein
FDA: US Food and Drug Administration
APS: Ammonium persulfate
TEMED: Tetramethylethylenediamine
PBS: Phosphate-buffered saline
FITC: Fluorescein isothiocyanate
FTIR: Fourier-transform infrared
TGA: Thermogravimetric analysis
XRD: X-ray diffraction
DMA: Dynamic mechanical analyzer
SR: Swelling ratio
DMEM: Dulbecco’s modified Eagle medium
H&E: Hematoxylin and eosin
