# Supplementary figures and images for: Synthesis of aligned porous polyethylene glycol/silk fibroin/hydroxyapatite scaffolds for osteoinduction in bone tissue engineering
Source: Stem Cell Res Ther. 2020 Dec 3;11:522. doi: 10.1186/s13287-020-02024-8 (PMC7712560; doi:10.1186/s13287-020-02024-8)

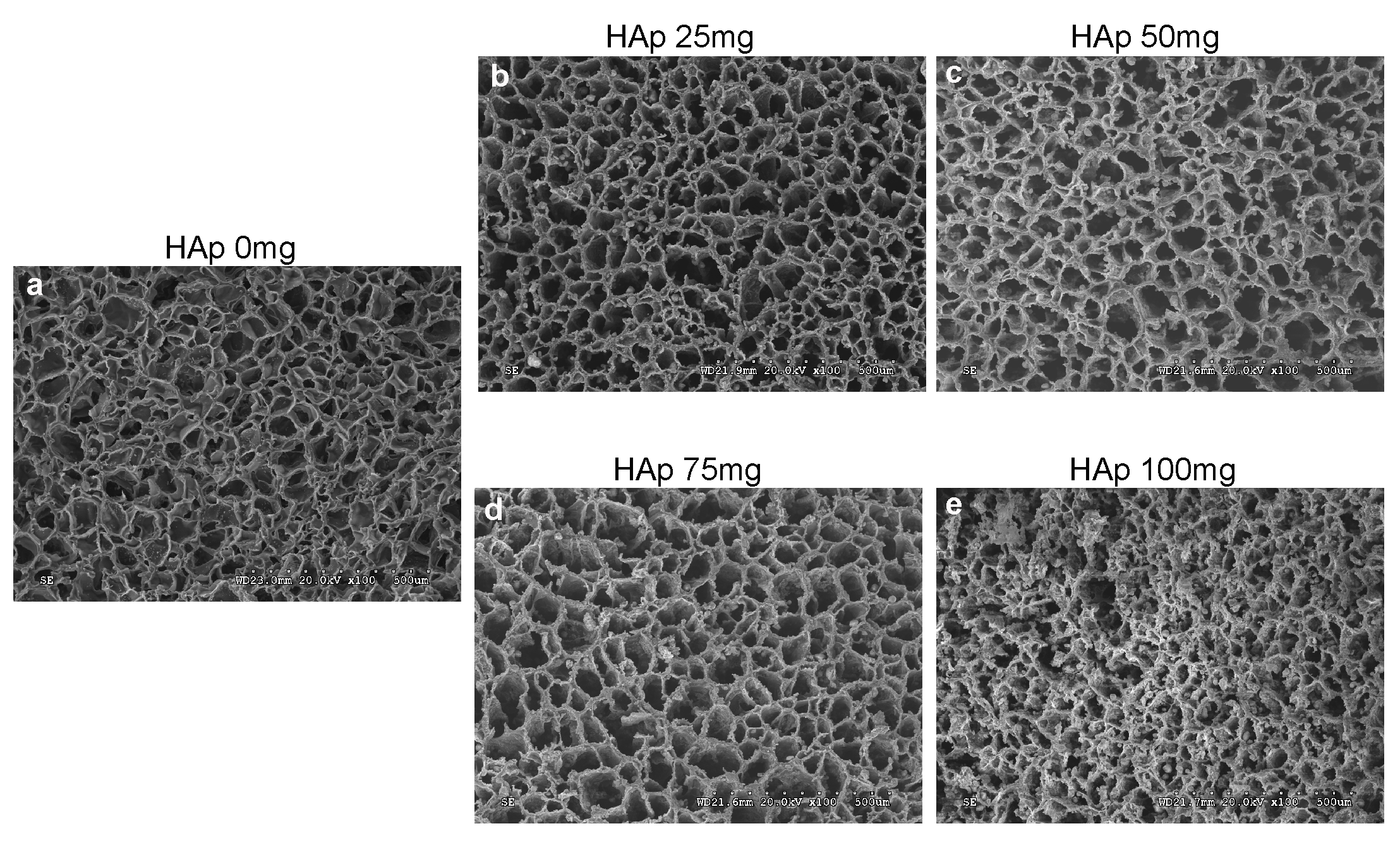

Supplement: Supplementary file 1 — Additional file 1: Figure S1. SEM images of the structure and morphology of the scaffolds without HAp and with different HAp concentrations (scale bar = 500 μm). [file 13287_2020_2024_MOESM1_ESM.tif]

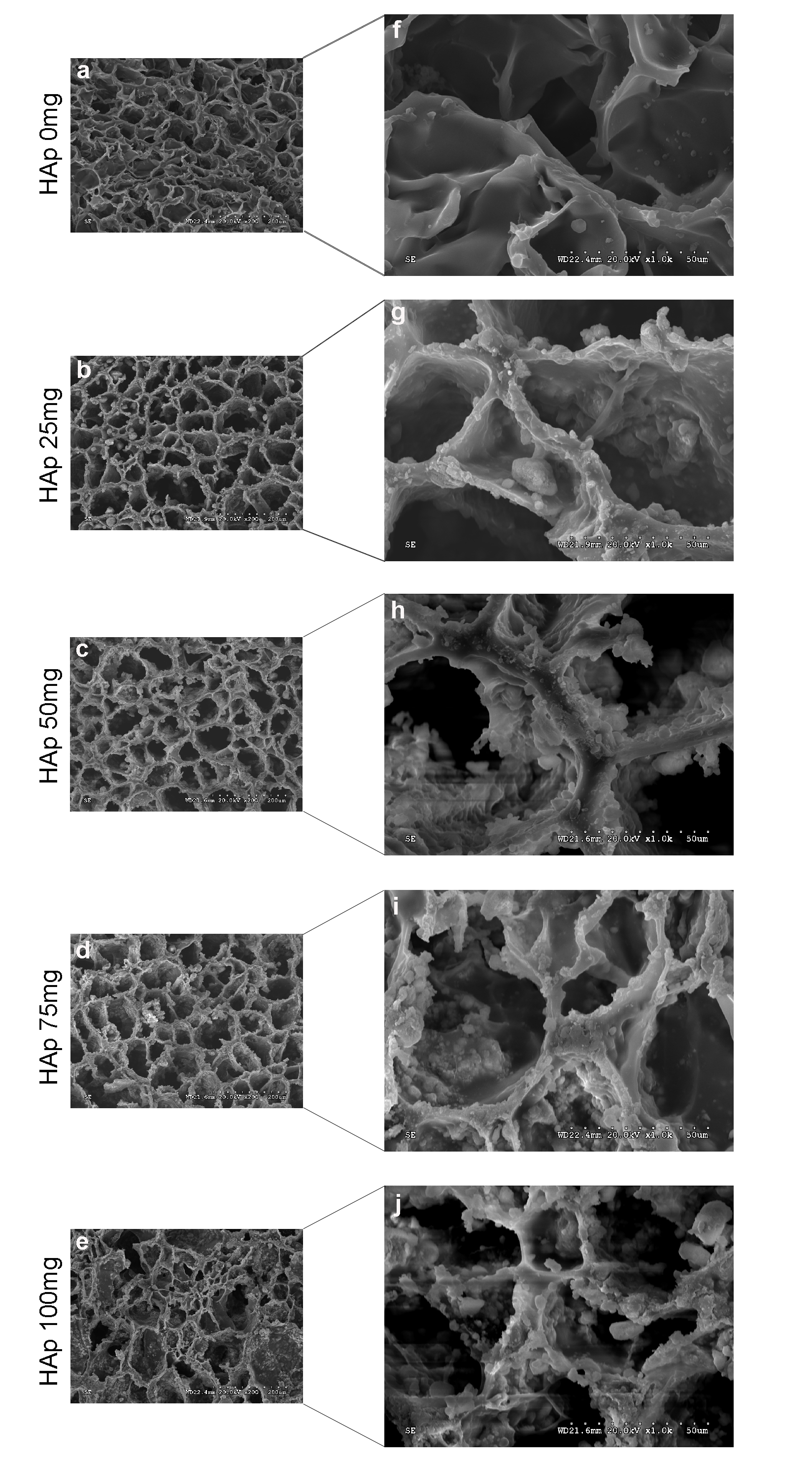

Supplement: Supplementary file 2 — Additional file 2: Figure S2. High-magnification SEM images (right) of the scaffolds without HAp and with different HAp concentrations. [file 13287_2020_2024_MOESM2_ESM.tif]

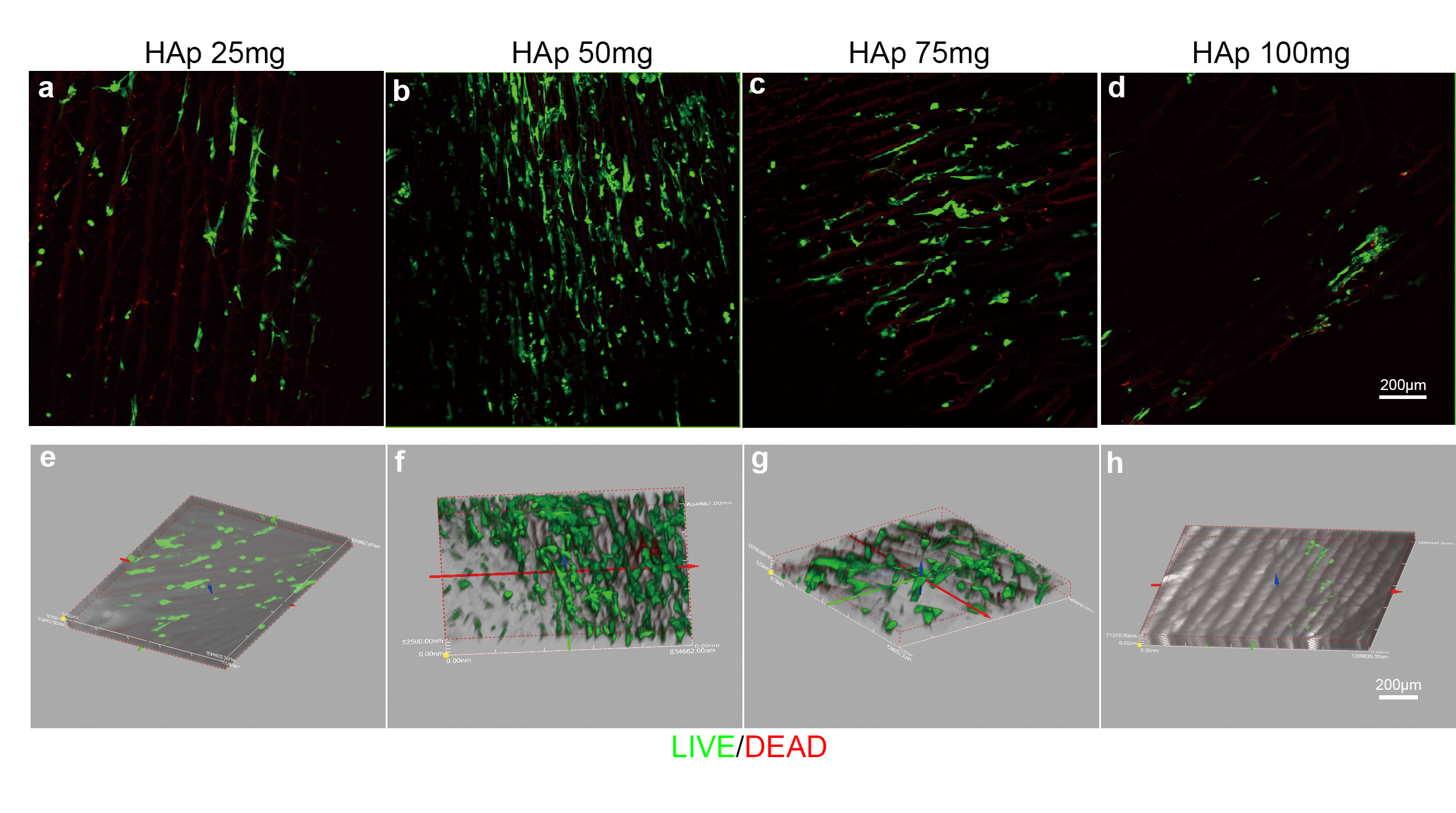

Supplement: Supplementary file 3 — Additional file 3: Figure S3. BMSC culture on the scaffold showing alignment during growth in the parallel section, based on the 2D view (a–d) and 3D view (e–h). (scale bar = 200 μm). [file 13287_2020_2024_MOESM3_ESM.tif]

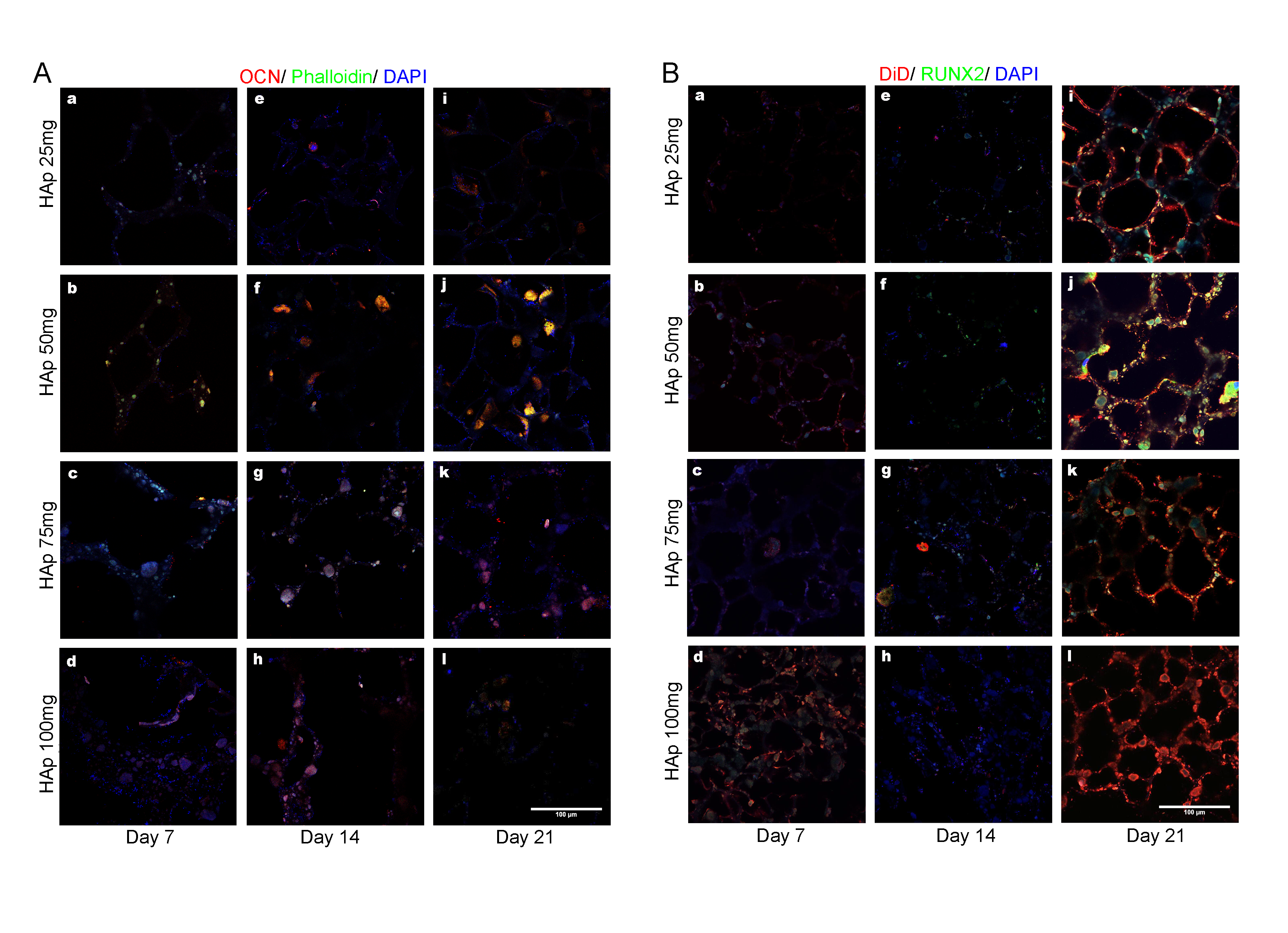

Supplement: Supplementary file 4 — Additional file 4: Figure S4. Immunofluorescence staining of osteocalcin (OCN) (A) and runt-related transcription factor 2 (RUNX2) (B) in BMSCs cultured for 7, 14, and 21 days on the scaffolds, based on the 2D view (scale bar = 100 μm). [file 13287_2020_2024_MOESM4_ESM.tif]

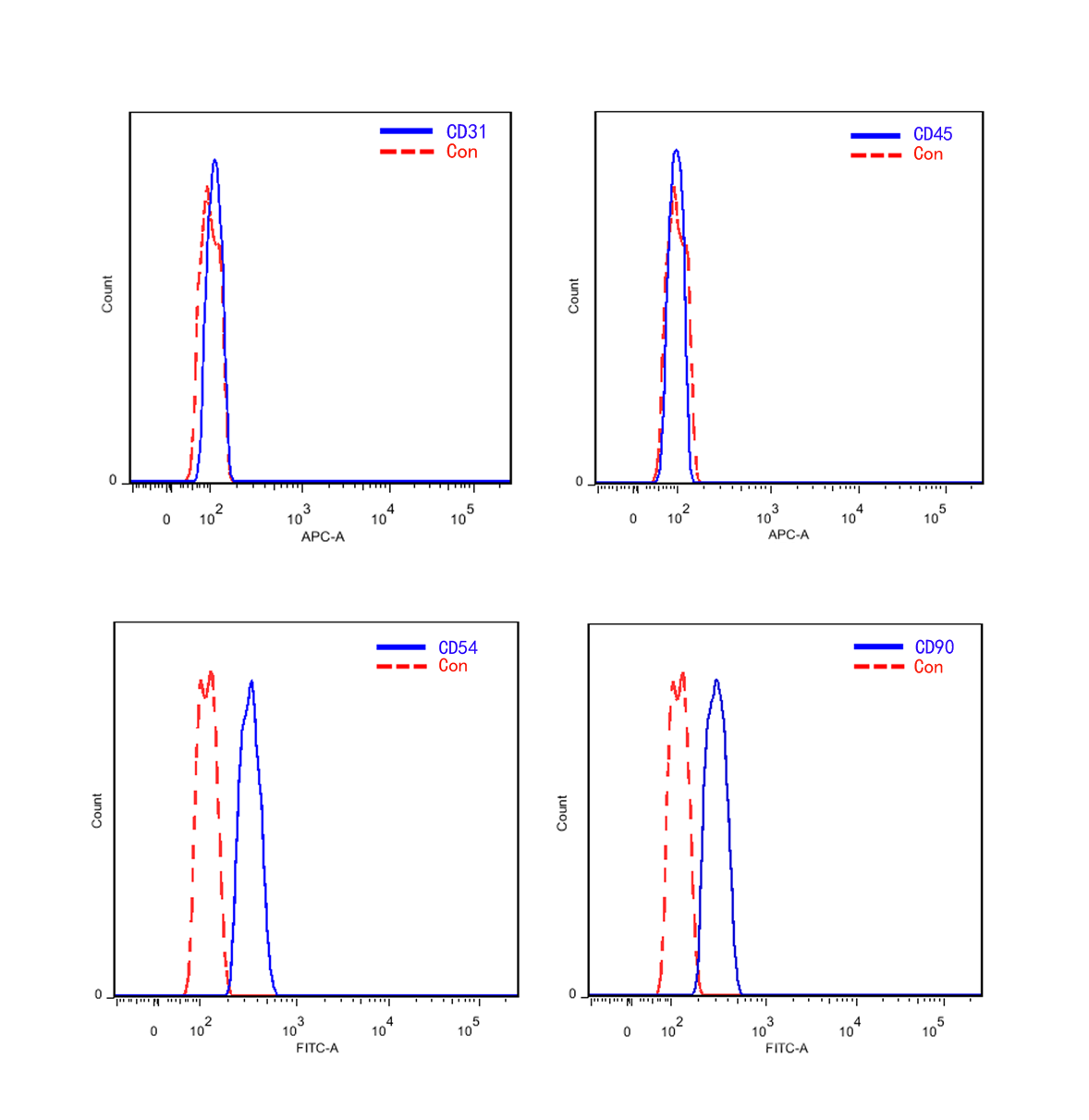

Supplement: Supplementary file 5 — Additional file 5: Figure S5. Surface molecular profile of BMSCs. [file 13287_2020_2024_MOESM5_ESM.tif]

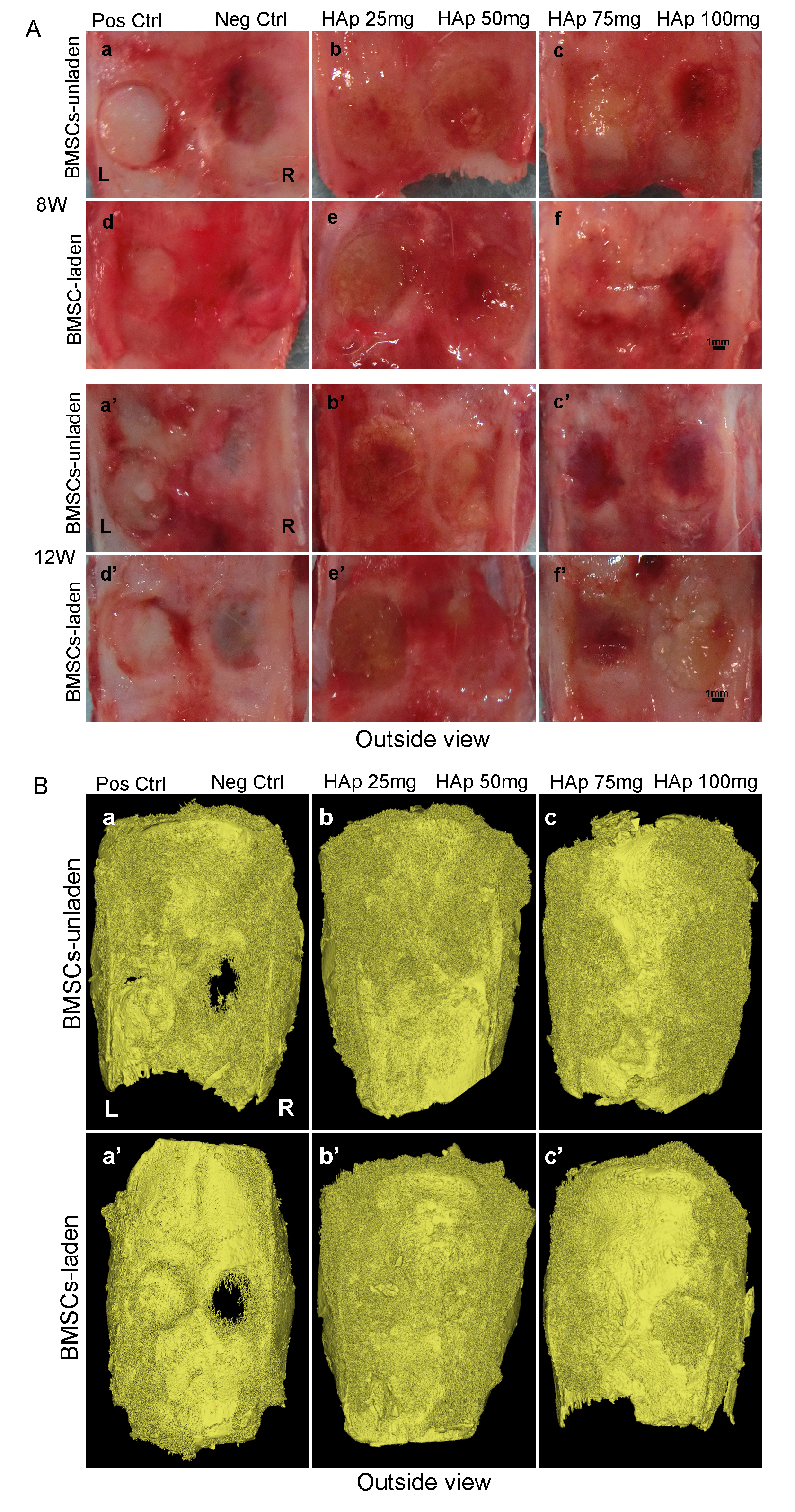

Supplement: Supplementary file 6 — Additional file 6: Figure S6. A. The morphology of the regenerated tissues in the calvarial defect area after 8 and 12 weeks (outside view, scale bar = 1 mm). a-c and a’-c’ were BMSCs-unladen positive control group, negative control group, HAp 25 mg group, HAp 50 mg group, HAp 75 mg group, and HAp 100 mg group respectively. d-f and d’-f’ were BMSCs-laden positive control group, negative control group, HAp 25 mg, HAp 50 mg, HAp 75 mg, and HAp 100 mg groups. B. Three-dimensional reconstruction images of micro-CT scanning data of the samples after 12 weeks of calvarial defect model by PEG/SF/HAp scaffold implanted with unladen (a-c) and laden (a’-c’) BMSCs (outside view). [file 13287_2020_2024_MOESM6_ESM.tif]

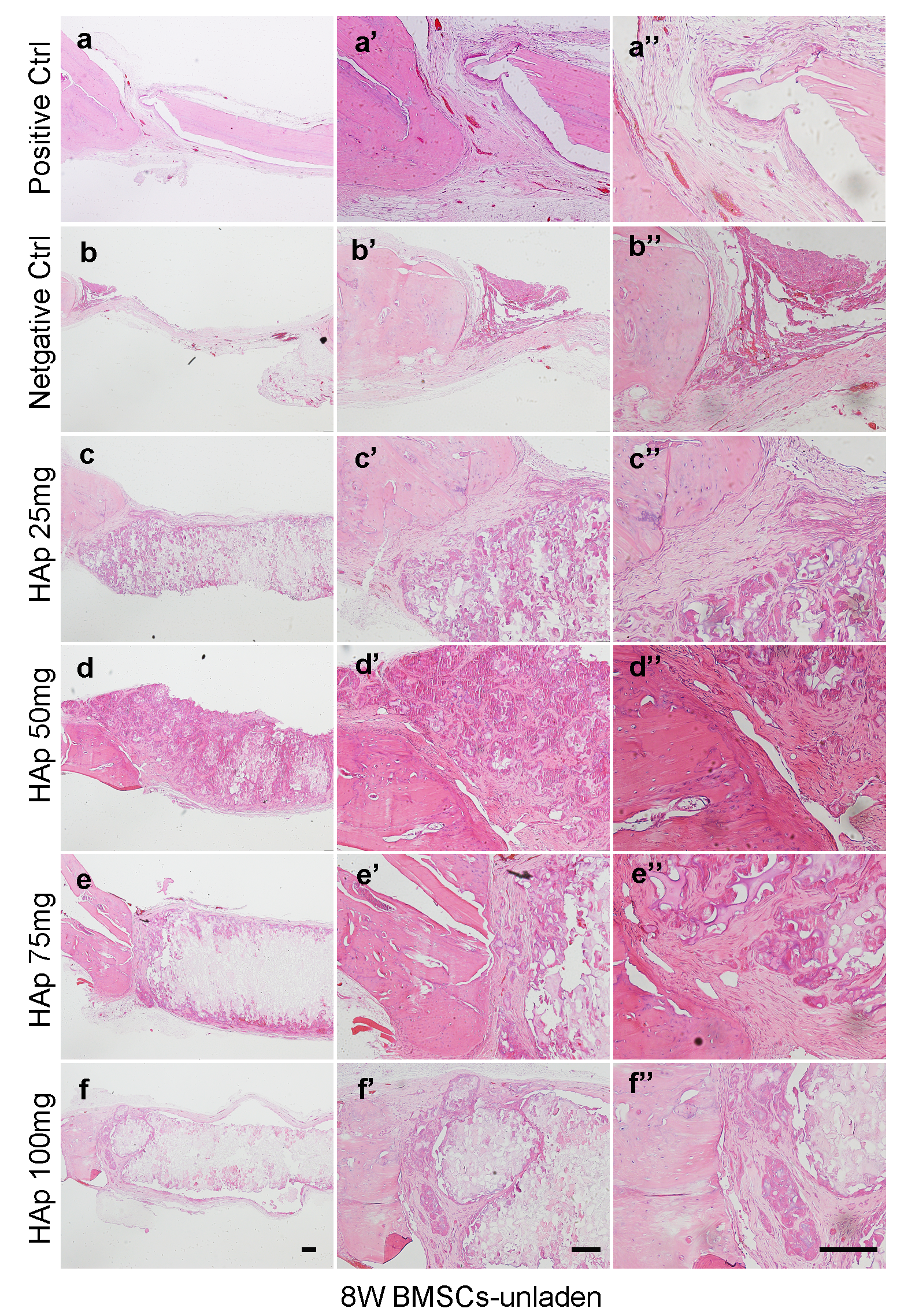

Supplement: Supplementary file 7 — Additional file 7: Figure S7. H&E staining of specimens in each group unladen with BMSCs at 8 weeks (scale bar = 200 μm). [file 13287_2020_2024_MOESM7_ESM.tif]

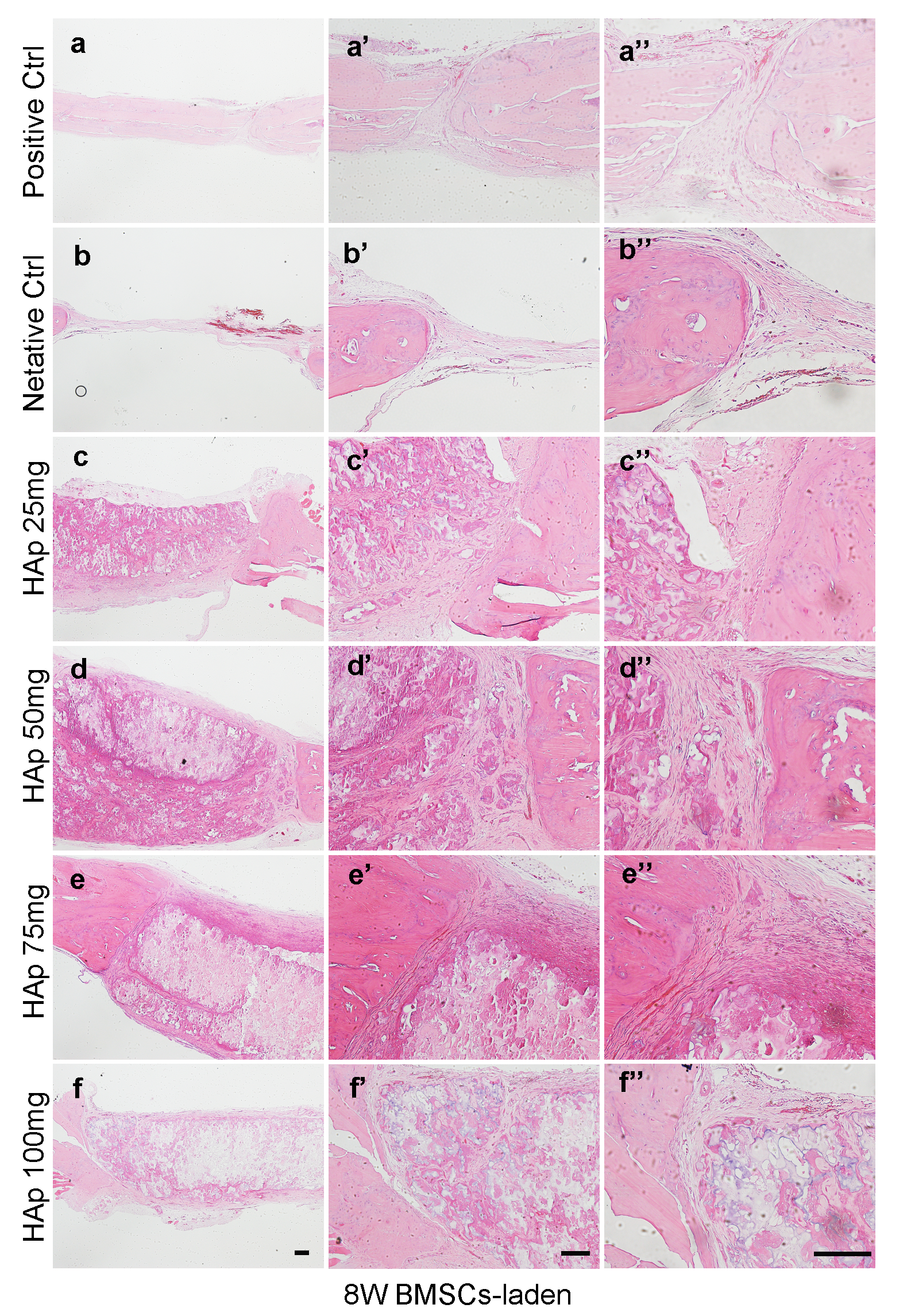

Supplement: Supplementary file 8 — Additional file 8: Figure S8. H&E staining of specimens in each group laden with BMSCs at 8 weeks (scale bar = 200 μm). [file 13287_2020_2024_MOESM8_ESM.tif]

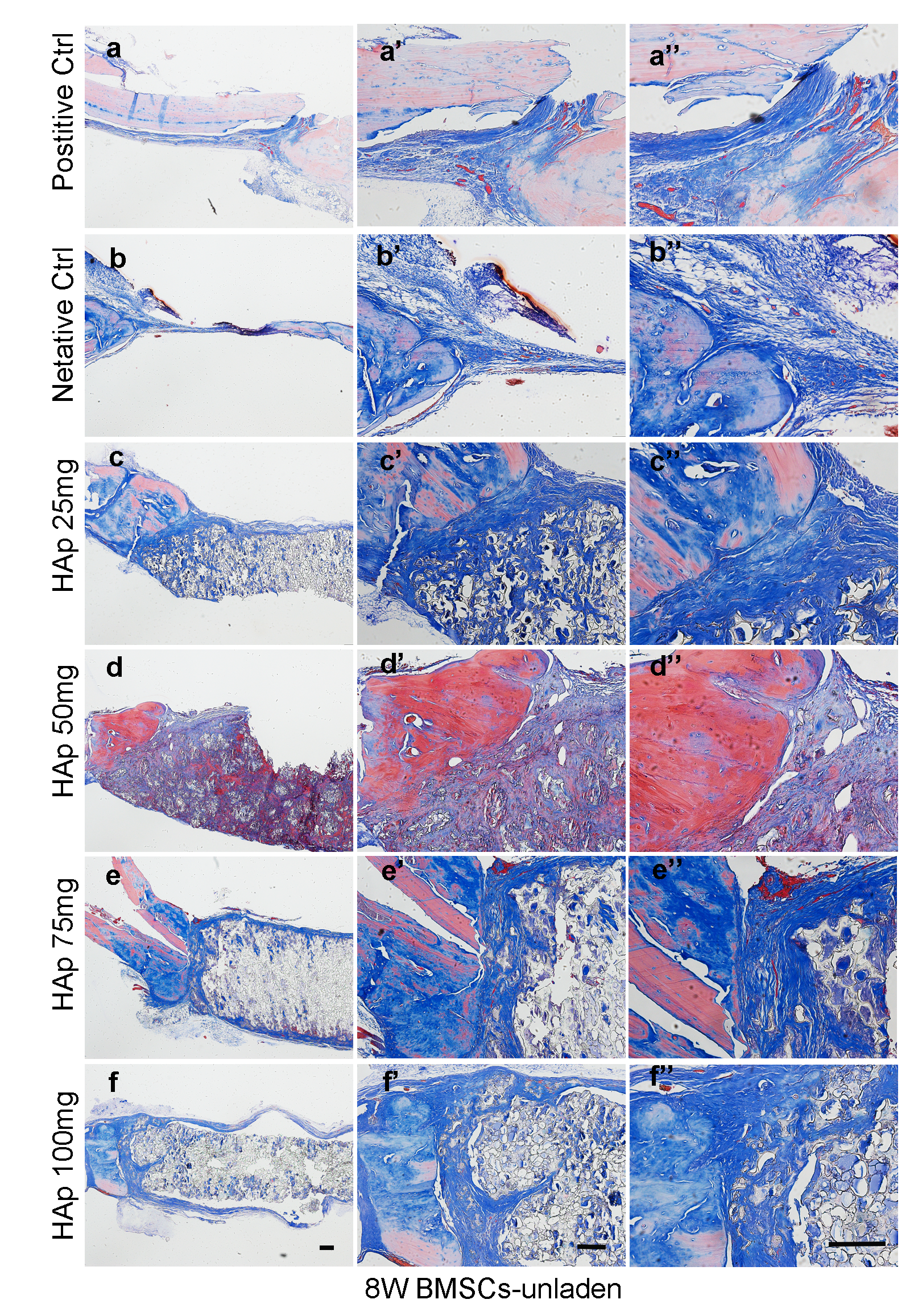

Supplement: Supplementary file 9 — Additional file 9: Figure S9. Masson trichrome staining of specimens in each group unladen with BMSCs at 8 weeks (scale bar = 200 μm). [file 13287_2020_2024_MOESM9_ESM.tif]

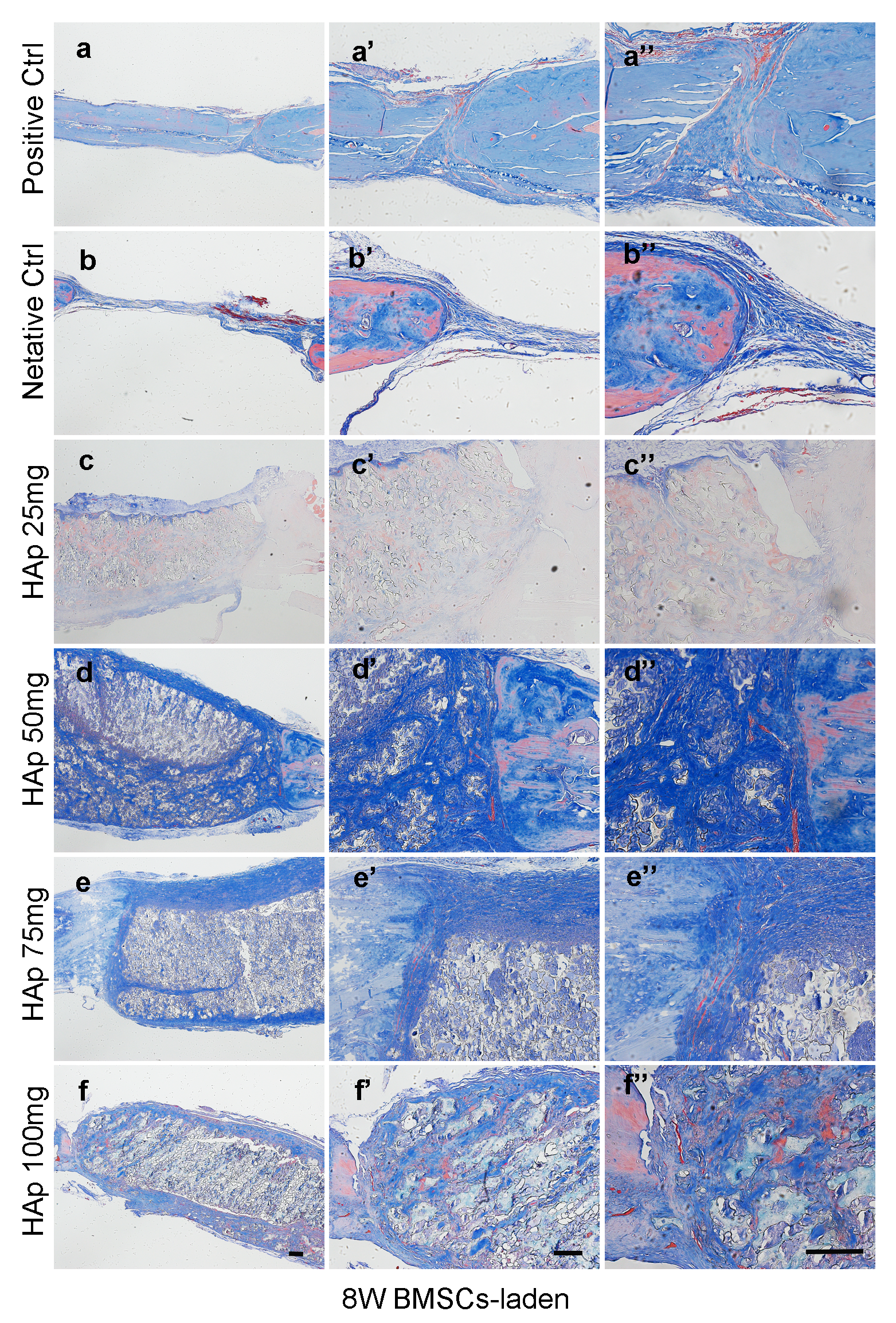

Supplement: Supplementary file 10 — Additional file 10: Figure S10. Masson trichrome staining of specimens in each group laden with BMSCs at 8 weeks (scale bar = 200 μm). [file 13287_2020_2024_MOESM10_ESM.tif]

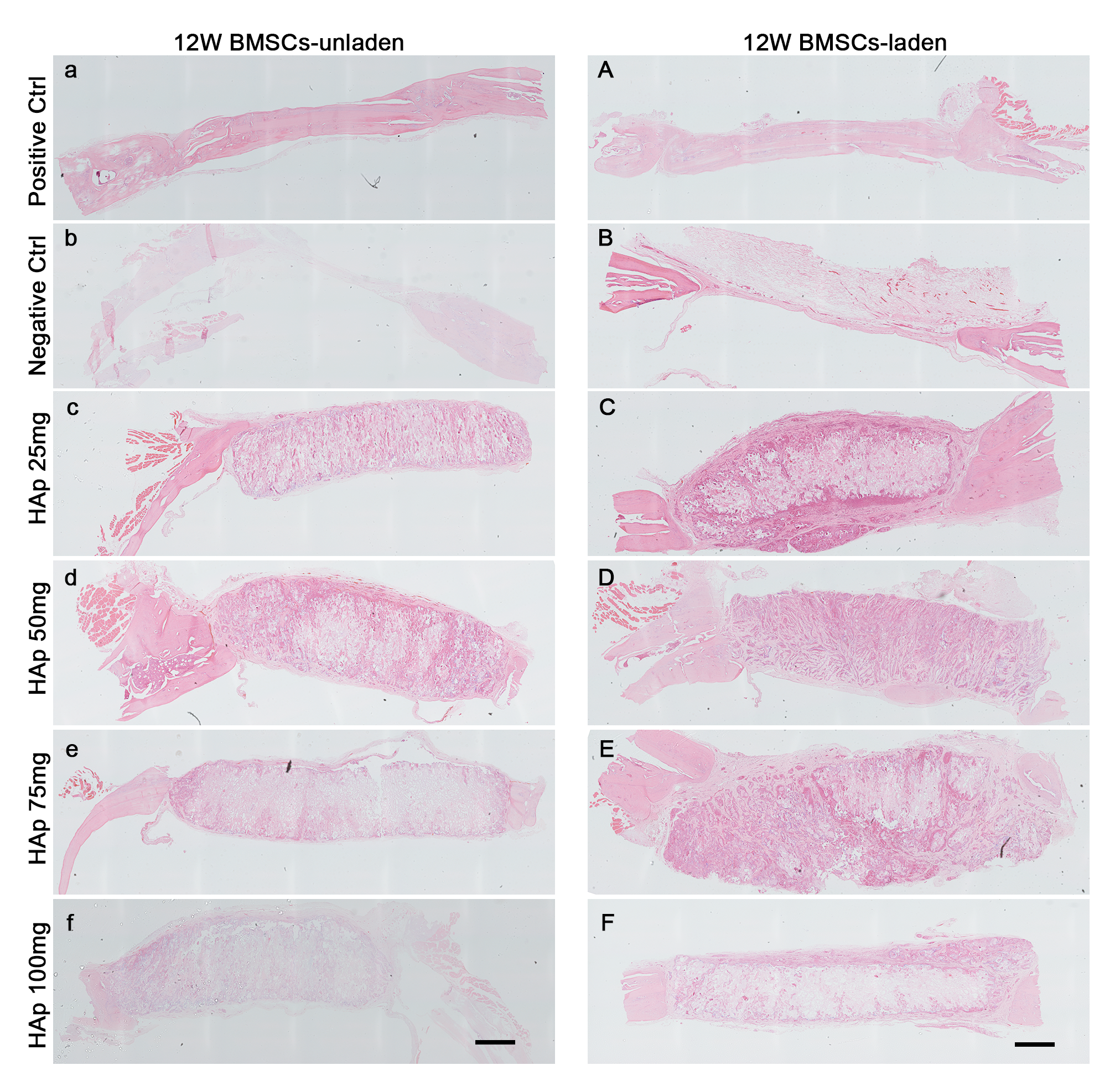

Supplement: Supplementary file 11 — Additional file 11: Figure S11. Full morphology of H&E staining of specimens in each group unladen and laden with BMSCs at 12 weeks (scale bar = 500 μm). [file 13287_2020_2024_MOESM11_ESM.tif]

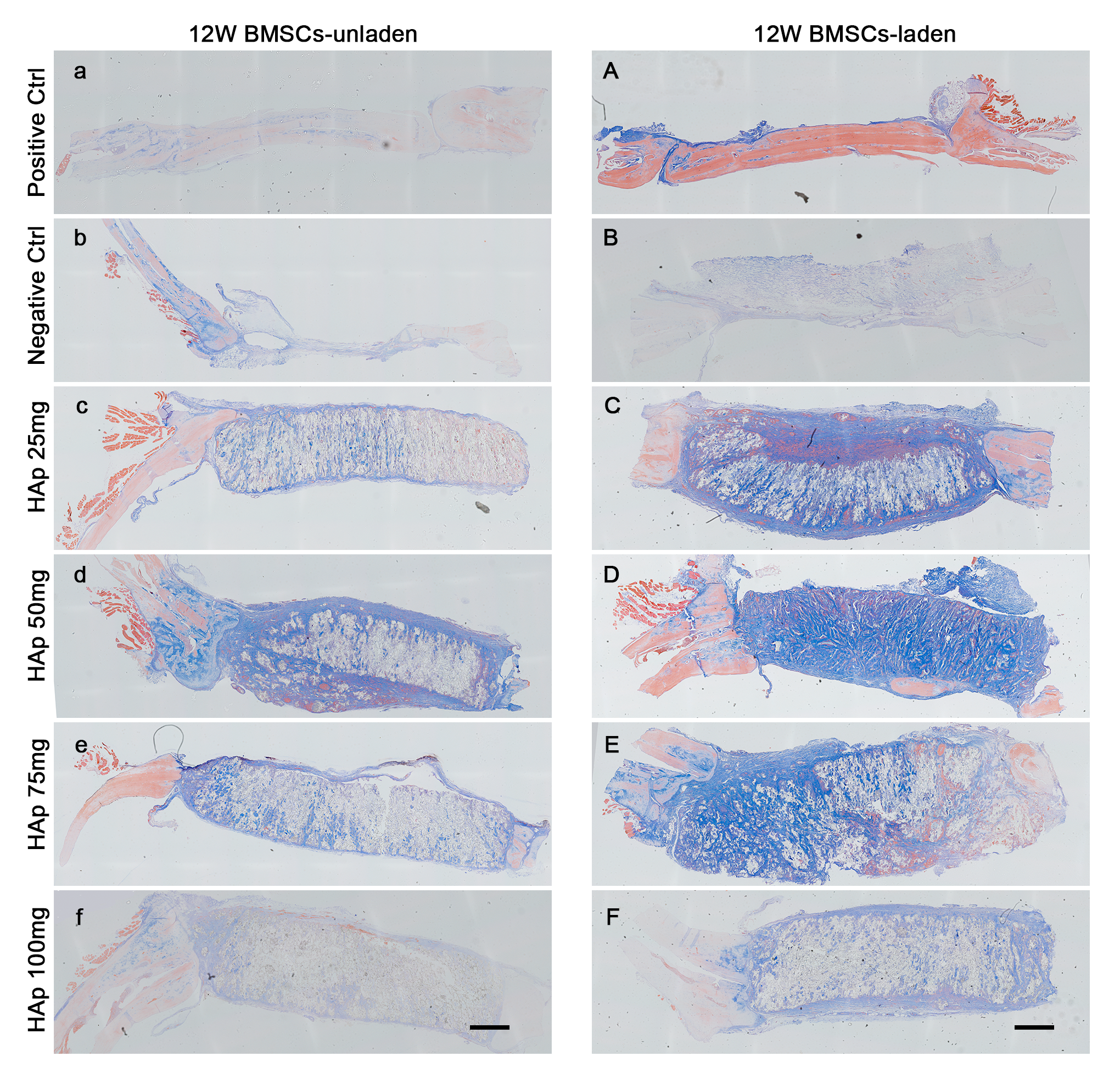

Supplement: Supplementary file 12 — Additional file 12: Figure S12. Full morphology of Masson staining of specimens in each group unladen and laden with BMSCs at 12 weeks (scale bar = 500 μm). [file 13287_2020_2024_MOESM12_ESM.tif]
