# Supplementary material for: Synthesis of aligned porous polyethylene glycol/silk fibroin/hydroxyapatite scaffolds for osteoinduction in bone tissue engineering
Source: Stem Cell Res Ther. 2020 Dec 3;11:522. doi: 10.1186/s13287-020-02024-8 (PMC7712560; doi:10.1186/s13287-020-02024-8)
Supplement: Supplementary file 17 — Additional file 17: Table S1. Young’s modulus of the scaffolds with different concentration of hydroxyapatite (HAp). [file 13287_2020_2024_MOESM17_ESM.docx]

Table S1. Young’s modulus of the scaffolds with different concentration of hydroxyapatite (HAp)

|  | **HAp 25mg** | **HAp 50mg** | **HAp 75mg** | **HAp 100mg** |
| --- | --- | --- | --- | --- |
| **Stiffness(kPa)** | 80.98±17.45 | 127.31±12.55 | 165.32±9.63 | 190.51±3.02 |
